# Methods in mol­ecular photocrystallography

**DOI:** 10.1107/S2053229624007460

**Published:** 2024-09-04

**Authors:** Lauren E. Hatcher, Mark R. Warren, Paul. R. Raithby

**Affiliations:** a School of Chemistry, Cardiff University, Main Building, Park Place, Cardiff, CF10 3AT, United Kingdom; b Diamond Light Source, Harwell Science and Innovation Campus Fermi Ave Didcot OX11 0DE United Kingdom; c Department of Chemistry, University of Bath, Bath, BA2 7AY, United Kingdom; University of Strathclyde, United Kingdom

**Keywords:** single-crystal X-ray diffraction, photocrystallography, time-resolution, synchrotrons, metastable mol­ecules, excited states, lifetimes, absorption spectra, pump-probe experiments, pump-multiprobe experiments, XFELs, lasers, LEDs

## Abstract

Over the last three decades, the technology that makes it possible to follow chemical processes in the solid state in real time has grown enormously. These studies have important implications for the design of new functional materials for applications in optoelectronics and sensors. Light–matter inter­actions are of particular importance, and *photocrystallography* has proved to be an important tool for studying these inter­actions. In this technique, the three-dimensional structures of light-activated mol­ecules, in their excited states, are determined using single-crystal X-ray crystallography. With advances in the design of high-power lasers, pulsed LEDs and time-gated X-ray detectors, the increased availability of synchrotron facilities, and most recently, the development of XFELs, it is now possible to determine the structures of mol­ecules with lifetimes ranging from minutes down to picoseconds, within a single crystal, using the photocrystallographic technique. This review discusses the procedures for conducting successful photocrystallographic studies and outlines the different methodologies that have been developed to study structures with specific lifetime ranges. The com­plexity of the methods required increases considerably as the lifetime of the excited state shortens. The discussion is supported by examples of successful photocrystallographic studies across a range of timescales and emphasises the importance of the use of com­plementary analytical techniques in order to understand the solid-state processes fully.

## Abstract 2

Over the last three decades, the technology that makes it possible to follow chemical processes in the solid state in real time has grown enormously. These studies have important implications for the design of new functional materials for applications in optoelectronics and sensors. Light–matter inter­actions are of particular importance, and *photocrystallography* has proved to be an important tool for studying these inter­actions.

## Background

The term *Photocrystallograpy* has been used since the 1990s to describe diffraction experiments in which the structure of a metastable or excited state species within a crystalline solid is determined by crystallography when the material is activated by light (Cole, 2008 ▸; Coppens, 2017 ▸). The term was first used by Philip Coppens (Carducci *et al.*, 1997 ▸), one of the pioneers in the research area, and is now used to refer to a wide range of experiments in which photochemical and crystallographic techniques are used to study dynamic processes in crystalline solids. An analogous term *X-ray photodiffraction* (Naumov, 2012 ▸) has also been used to describe experiments in which diffraction has been used to study the salient effects of dynamic crystals which undergo a rapid phase transition upon exposure to light (photo-salient), causing the crystalline sample to rapidly move or in some cases jump (Naumov *et al.*, 2020 ▸). Heat (thermo-salient) or localized pressure (mechano-salient) effects can also initiate rapid movement in crystalline samples.

*Photocrystallography* brings the dimension of time into the crystallographic experiment (Raithby, 2007 ▸). In a conventional single-crystal X-ray crystallographic experiment, while the inter­action of a single X-ray photon with the electron density is very fast, of the order of 10^−18^ s, in order to solve and refine the crystal structure, the collection of the intensities from all the reflections (Bragg peaks) is required and results in an overall experiment time on a scale of minutes to hours. The resulting three-dimensional (3D) structure is, therefore, both a time average across the total experiment and a space average over the whole diffracting crystal. The development of time-resolved diffraction techniques, coupled with dramatic advances in numerous contingent technologies, including the increases in intensity and brightness of X-ray sources, the rapidity and accuracy of modern X-ray detectors, improvements in cryogenics and laser technology, and increased com­puting power and data storage capacity, has changed the situation hugely. These advances have opened up the field of Time-Resolved Single-Crystal X-ray Diffraction (TR-SCXRD), allowing the use of 3D X-ray methods to study dynamic processes as they occur in crystalline materials, with applications across the physical and life sciences.

Time-resolved crystallography was developed for macromolecular systems before mol­ecular systems because of the inter­est in and importance of many biological processes. Biological crystals are more susceptible to X-ray damage than mol­ecular crystals, so faster data collection strategies are beneficial to minimize this effect. As a result, the first macromolecular studies were carried out using Laue diffraction techniques, where the crystal is irradiated with a continuum of X-ray wavelengths from a polychromatic, or ‘white’, X-ray beam (Drenth, 2007 ▸). This methodology makes more efficient use of the X-ray beam, with many more reflections being collected in a single diffraction image than is possible with monochromatic radiation. However, experimental techniques needed to be developed to reach the short timescales required to study the biological processes of inter­est.

In 1996, Moffat and co-workers reported the first light-induced nanosecond-resolved crystallographic investigation on the photodissociation mechanism of CO in carbon mon­oxy-myoglobin (MbCO) (Šrajer *et al.*, 1996 ▸). The reaction in solution had already been studied in detail using ultra-fast spectroscopic methods (Lim *et al.*, 1995 ▸), and in the diffraction study, Moffat and co-workers showed that the diffraction data correlated well with the solution studies. Using a pump–probe strategy, they found that, with probe delays (τ) of between 4 ns and 1 µs after the initial pump (activation) pulse, there was a reduction in electron density at the expected position of the CO ligand bound to the Fe centre. This indicated that photoactivation of the CO had occurred causing the CO to dissociate from the metal centre and begin to move away, within the confines of the crystalline environment. In a later study by the same group, with probe delays of between τ = 1 ns and 1 µs, the positions of transient docking sites for the photodissociated CO ligand were identified, and the pathway for the photodissociation process identified (Srajer *et al.*, 2001 ▸). Since these pioneering studies, there has been an explosion in investigations into the dynamic structures and reactivity of proteins and other macromolecules using syn­chro­trons and X-ray free electron lasers (XFELs) (Spence *et al.*, 2012 ▸; Chapman, 2019 ▸; Levantino *et al.*, 2021 ▸).

For mol­ecular systems, photoinduced changes in crystals have been studied since the 1960s, well before the term *photocrystallography* was coined. Schmidt and Cohen studied the irreversible [2 + 2] photodimerization within crystals of a series of *trans*-cinnamic acid derivatives, using sunlight to photoactivate them (Cohen & Schmidt, 1964 ▸; Cohen *et al.*, 1964 ▸; Schmidt, 1964 ▸), and powder and single-crystal X-ray dif­fraction to monitor the dimerization. The authors analysed the crystalline environment in which the dimerization oc­curred. They highlighted a set of criteria that must be satisfied if single-crystal-to-single-crystal transformations were to occur. They put forward their *Topochemical Postulate* stating that ‘*the photoreaction will follow a minimum energy pathway that imparts the lowest level of steric strain on the surrounding crystal array*’. While there have been subsequent improvements (Cohen, 1975 ▸) and the identification of some exceptions (Natarajan & Bhogala, 2011 ▸), the *Topochemical Postulate* remains an effective guideline for designing systems that will undergo high levels of solid-state photoconversion.

Building on this *Postulate* and by examining single-crystal-to-single-crystal reactions and their kinetics, Ohashi proposed the idea of a *reaction cavity* (Ohashi *et al.*, 1981 ▸). Since, for a single-crystal-to-single-crystal transformation to occur, the crystal integrity must be maintained, it follows that there must be a void space around the reactive groups that allows the required movement without disrupting the wider crystalline lattice. It further follows that the available void space essentially controls the reactivity of the crystal. In his pioneering work on a series of cobaloxime com­plexes, Ohashi showed that the *reaction cavity* displayed flexibility through the process of the reaction and that changes in tem­per­a­ture could significantly affect the reaction, lowering the tem­per­a­ture and causing lattice contraction, reducing the *cavity* size and thus terminating the process (Ohashi, 2013 ▸). These ideas can readily be related to the types of inter­molecular inter­actions present within the crystalline environment, including hydrogen bond­ing and π–π stacking inter­actions, and *Crystal Engineering* concepts have proved helpful in the inter­pretation of *photocrystallographic* experiments (Hatcher, Bigos *et al.*, 2014 ▸; Naumov *et al.*, 2020 ▸; Hatcher *et al.*, 2023 ▸).

## Planning a single-crystal *photocrystallographic* experiment

When planning a *photocrystallographic* experiment on a mol­ecular crystal system, there are a whole series of contributing factors to consider. Some of these factors are associated with the mol­ecular photochemistry of the material being studied and others are associated with its crystal structure. What has been learned is that it is unwise to attempt a photocrystallographic experiment unless a whole range of com­plementary experimental and com­putational techniques have been applied to the chemical process and its nature in the crystalline state is well understood.

### Complementary techniques

Firstly, when considering the photochemistry of the molecule, it is important to establish the nature of the reaction process, the reaction products, the wavelengths of light that cause the photoactivation, the timescale of the reaction and the quantum yield of the reaction. These may already be known for the process in solution, but we are specifically considering the solid-state (preferably crystalline-state) pro­cess. That process may not be the same as is observed in solution. If the spectroscopic studies have not already been determined for the solid-state material, they should be carried out as a first step. Spectroscopy and com­putational investigations might include:

– A full photophysical analysis (optical absorption spectroscopy/fluorescence spectroscopy) will provide information on the photochemistry of the crystals.

– Time-averaged absorption spectra can inform the choice of irradiation wavelength to be used. When exciting a crystal with light, it is prudent not to irradiate the crystal at the absorption maximum (λ_max_) because the size of the crystal may exceed the penetration depth of the light at that wavelength, that is, the light beam may be stopped within a few nanometres of the crystal surface and mol­ecules deeper in the crystal will not be excited, limiting the level of conversion possible. In crystals, it is better to irradiate with a wavelength of light that is within the absorption peak but is half to two-thirds towards the absorption maximum in the tail of the band, as this radiation will penetrate more deeply into the crystal (Enkelmann *et al.*, 1993 ▸). If there is an overlap of the ab­sorp­tion of the ground state and photoexcited state species, then, although the reactant will be excited, driving the reaction forward, the product formed may also absorb the excitation light and simultaneously drive the back reaction. In this situation, an equilibrium will be set up, and achieving com­plete conversion to the forward reaction product will not be possible.

– Time-resolved optical (absorption/fluorescence) and vi­bra­tional (Raman/IR) spectroscopy will provide information on excited state lifetimes for fast pump–probe experiments.

– A com­prehensive com­putational study of the reaction process may point to the formation of likely reaction products or identify side reactions that may occur.

### Suitability of the crystals

For a single-crystal photocrystallographic experiment to be successful, it is essential that the quality of the crystal being studied is high. It should display a clean diffraction pattern to better than atomic resolution for the ground state structure. Preliminary crystallographic data collections across a range of tem­per­a­tures are necessary to optimize the data collection parameters for the *photocrystallographic* experiments, and to highlight any unexpected effects, such as phase transitions. Other important factors to consider include:

– Crystal size: small crystals are preferred for *photocrys­tallography* studies to maximize light penetration, as men­tioned previously.

– Crystal shape: for irradiation experiments involving lasers, illumination along specific crystal directions/normal to specific faces can be important. It is helpful to face-index the crystal to understand the crystal morphology prior to irradiation.

– Crystal system/space group: the crystal symmetry will affect the amount of unique data required and, thus, the speed of the experiment; this is especially important for stroboscopic pump–probe experiments.

– Diffraction power: small crystals often require high-flux X-rays (*e.g.* synchrotron radiation) to collect X-ray data in a reasonable time.

– Crystal stability: if the crystals are air or moisture sensitive precautions may have to be taken during the *photocrystallographic* experiment to avoid deterioration. If the crystals are sensitive to changes in tem­per­a­ture and undergo phase transitions or display hysteretic behaviour, the tem­per­a­ture range over which variable-tem­per­a­ture photocrystallographic studies can be carried out may be limited.

### Factors involving the crystal structure

The way in which the mol­ecules crystallize within the crystal may influence the path of the photoactivated process, and this is an additional constraint when com­pared to photochemical reactions in solution. Factors to consider include:

– Large changes in unit-cell parameters during the photoactivation may cause crystal deterioration to occur so that reversible single-crystal-to-single-crystal transformations are not possible. There are few examples of reversible photoactivation experiments where the unit-cell parameters change by more than 3%. Often, this problem can only be identified during the course of the photocrystallographic experiment, but the choice of chemical systems where there is flexibility within the crystal structure can prevent this problem, as highlighted by Ohashi with his reaction cavity concept (Ohashi, 2013 ▸). The use of metal–organic frameworks (MOFs) that act as cages to enclose the photoactivated species can be helpful (Blake *et al.*, 2010 ▸), or simply the use of bulky auxiliary ligands or the presence of bulky counter-ions in com­plex salts that create void space within the crystal structure.

– Inter­molecular inter­actions within the crystal can either facilitate or block the progress of a solid-state reaction. It is hard to predict what the combined effect of a particular set of inter­molecular inter­actions is, but there are several examples in the photocrystallographic literature where there are crystals with two crystallographically independent but chemically equivalent mol­ecules in the crystallographic asymmetric unit that behave somewhat differently under photoactivation, and these differences are attributed to the presence of different inter­molecular inter­actions (Coppens *et al.*, 2013 ▸; Hatcher, Bigos *et al.*, 2014 ▸).

Only after considering all of the points in the previous three sections and their implications is it sensible to proceed with a single-crystal *photocrystallographic* experiment. If at all pos­sible, it is beneficial to simultaneously measure spectroscopic data from the crystal while the photocrystallographic measurements are being carried out so that the two forms of analysis can be correlated (Hasil *et al.*, 2024 ▸).

## Case histories

Before discussing the details of ‘*how to carry out a mol­ecular photocrystallographic experiment*’, it is helpful to describe some of the *photocrystallographic* experiments that have been undertaken successfully to provide the reader with an idea of what can currently be achieved, how the methodology is con­tinuing to develop and what the main challenges involved are. This is best achieved by describing a series of case histories.

**Figure 1:**
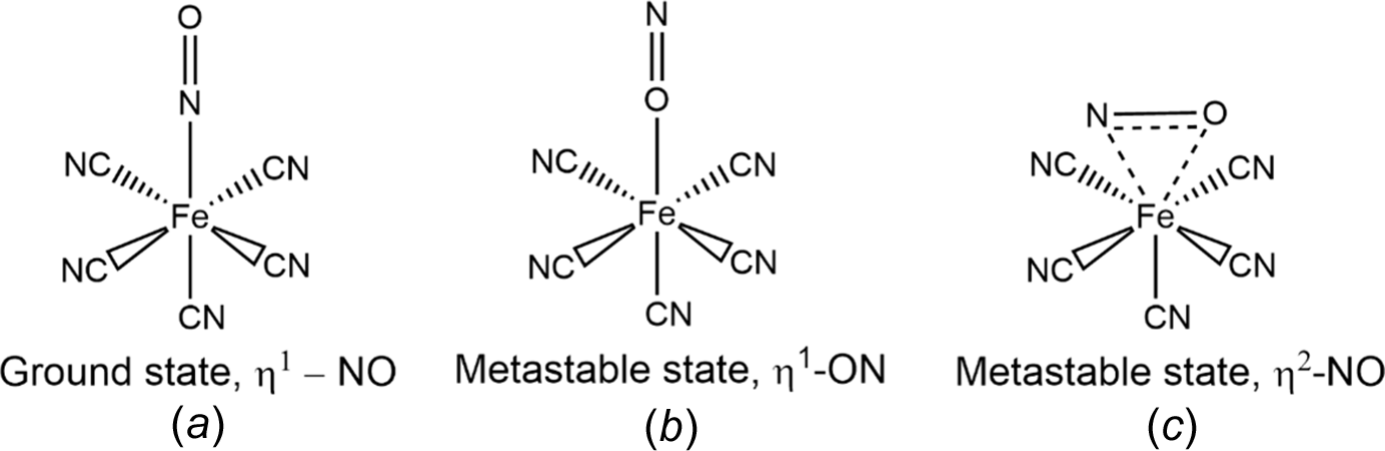
Structural diagram of the nitrosyl linkage isomers found in Na[Fe(CN)_5_(NO)] in the ground state and upon photoexcitation: (*a*) [Fe(CN)_5_(η^1^-NO)]^−^, (*b*) [Fe(CN)_5_(η^1^-ON)]^−^ and (*c*) [Fe(CN)_5_(η^2^-NO)]^−^.

Building on the results obtained for the irreversible solid-state photochemical reactions and taking into account the factors outlined above that determine whether or not a *photocrystallographic* experiment is likely to be successful, Coppens carried out the first studies on reversible mol­ecular systems in the 1990s (Pressprich *et al.*, 1994 ▸). Using steady-state crystallographic methods, Coppens showed that the solid-state photoactivation of single crystals of iron–nitrosyl com­plexes resulted in the conversion of the mol­ecule to a new *metastable* linkage isomer (Carducci *et al.*, 1997 ▸). In these crystallographic experiments, the term metastable is taken to mean that the structure of the mol­ecule being studied does not change during the duration of the data collection; in this instance, each data collection took up to 20 h using a rotating-anode X-ray generator and an image-plate diffractometer. Crystals of sodium nitro­prusside were irradiated with light from an Ar^+^ laser at λ = 488 nm and 50 K, until a *photostationary* state was reached. With irradiation, the nitrosyl coordination mode changed from a ground state (GS) η^1^-NO isomer to an excited state (ES) iso­nitrosyl η^1^-ON isomer with 37% population. The photoactivated crystal was then irradiated with 1064 nm laser light. With this irradiation, a second *metastable* linkage isomer was obtained, with the iso­nitrosyl isomer converting to a side-on-coordinated η^2^-NO isomer, with 10% population (Fig. 1 ▸). As is common in *photocrystallographic* experiments, because the conversion from one form to another is not 100% com­plete, the various structural forms must be refined as com­ponents of a disordered structural model with variable atomic occupancies (summing to unity) for the multiple com­ponents. In this example, in the case of the ground state η^1^-NO form and the iso­nitrosyl η^1^-ON form, there is considerable overlap of the N and O atoms in the two isomers, and the occupancies are best determined by corroborating experimental techniques rather than by trying to refine the occupancies within the crystallographic model. The value of 37% for the conversion to the iso­nitrosyl η^1^-ON isomer was obtained from supporting dif­ferential scanning calorimetry (DSC) studies (Woike *et al.*, 1993 ▸); however, because there is no atomic overlap between the η^1^-NO and η^1^-ON forms and the η^2^-NO isomer, the occupancy of the η^2^-NO group can be refined successfully within the crystallographic model, giving a value of 10% for this isomer.

In the photocrystallographic study, it was clear that it was not just the position of the nitrosyl group that changes upon excitation, but all the atoms in the structure. For example, for the [Fe(CN)_5_(η^2^-NO)]^−^ excited state isomer, the Fe atom and the *trans*-cyanide group are displaced towards the η^2^-NO ligand. Additionally, the disorder present limits the accuracy of the bond parameters associated with the nitrosyl group, and restricts any assessment of the bonding within the unit. Generally, in refinements of disordered groups, it may be necessary to apply constraints or restraints to atomic positions or bond parameters to obtain a stable refinement (see further discussion in Section 4.1 below). Clearly, if a bond parameter is restrained or constrained, nothing can be said about parameter changes upon excitation, and if the atoms are being refined freely, careful consideration needs to be given to the magnitude of the estimated standard deviations (e.s.d.’s) before changes in bond parameters can be evaluated meaningfully.

The use of DSC techniques to corroborate the metastable state occupancy in the example above reaffirms the importance of having a plethora of supporting information before undertaking even the most straightforward *photocrystallographic* experiments. In his 1997 article reporting the *photocrystallographic* study on sodium nitro­prusside (Carducci *et al.*, 1997 ▸), Coppens also either reported or drew upon pre­viously published Mössbauer, IR and Raman, and EPR spectroscopic studies, single-crystal X-ray and neutron dif­fraction studies of the ground state, and the observed colour change in the crystal when going from the ground to the metastable state supported by UV–visible (UV–Vis) spectroscopy, in order to plan the experiment and inter­pret the results. Quantum chemical calculations further underpinned the study. A subsequent neutron diffraction study also confirmed the nature of the two excited state nitrosyl isomers (Schaniel *et al.*, 2006 ▸). In much more recent studies on the nitrosyl linkage isomers of [Ru(py)_4_F(NO)](ClO_4_)_2_, where the differences and similarities of using either continuous wave (CW) or pulsed lasers to photoactivate the crystal, *in-situ* UV–Vis absorption spectroscopy has been used to monitor the conversion of the ground state to the excited state structure. IR spectra of KBr pellets of the com­plex have also been recorded before, during and after laser irradiation under careful tem­per­a­ture control to monitor the nitrosyl stretching frequencies and correlate them with the simultaneous X-ray and UV experiments (Hasil *et al.*, 2024 ▸).

**Figure 2:**
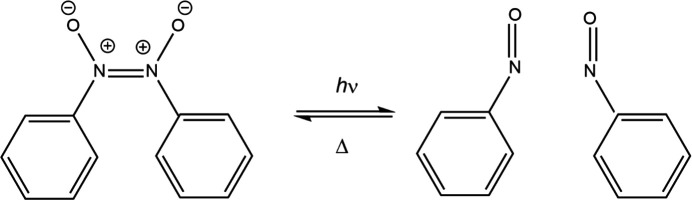
The *photocrystallographic* photolysis and redimerization of the *cis*-dimer of nitroso­benzene.

After the early groundbreaking work, there have been many *photocrystallographic* studies on the inter­conversion between linkage isomers in transition-metal com­plexes. The ligands studied include nitro­syls, nitrites, sulfur dioxide, di­nitro­gen and dimethyl sulfoxide (Fomitchev *et al.*, 2000 ▸; Coppens *et al.*, 2002 ▸; Casaretto *et al.*, 2015 ▸; Coppens, 2017 ▸; Schaniel *et al.*, 2018 ▸; Hatcher *et al.*, 2019 ▸; Cole, Gosztola, Velazquez-Garcia *et al.*, 2021 ▸; Borowski *et al.*, 2022 ▸; Cole *et al.*, 2022 ▸; Marr *et al.*, 2023 ▸; Mikhailov *et al.*, 2023 ▸; Potempa *et al.*, 2023 ▸). In most examples studied where photoactivated linkage isomerism occurs through a single-crystal-to-single-crystal transformation, there is no change in the crystallographic space group through the process, and the unit-cell volume alters by less than 3%. This observation reinforces the significance of the ideas relating to the *Topochemical Postulate* and the *reaction cavity* initially put forward by Cohen & Schmidt (1964 ▸) and Ohashi (2013 ▸), res­pectively, for solid-state processes. A recent *photocrys­tal­lo­graphic* study on the *cis*-nitroso­benzene dimer shows that, under irradiation with a mercury lamp for 20 minutes at 100 K, it undergoes a transformation to a pair of monomers with an 8.6% monomer population and then reversibly recombines (Fig. 2 ▸) (Rodenbough *et al.*, 2018 ▸). The two N atoms move by a remarkable 2.97 (5) Å during this process. Despite this large change in the local environment about the nitroso moieties, there is still only a minimal change in the unit-cell volume before and after irradiation, and no change in the space group, which tends to indicate this study still conforms to aspects of the *Topochemical Postulate*. It is likely that the com­paratively low excited state population may result from the limitation of maintaining crystal integrity, and if >10% of the mol­ecules were to excite, this may result in the degradation of the single-crystal environment. However, this study is noteworthy as it suggests that it may be possible to extend photocrystallographic studies to processes involving more substantial movement of atoms.

One of the driving forces for the investigation of mol­ecular systems that switch between ground and *metastable* states under light irradiation in the solid state is their application as mol­ecular switches in optoelectronic devices (Cole, 2011 ▸). To achieve this aim, it is helpful for the switching to be 100% efficient, with the crystalline product not containing any residual ground state structure or multiple isomeric *metastable* state structures, and for the switching process to occur near room tem­per­a­ture. During the last two decades, research efforts have focused on designing 100% efficient solid-state switching materials based on linkage isomers using the previously discussed photochemical and crystal engineering factors.

**Figure 3:**
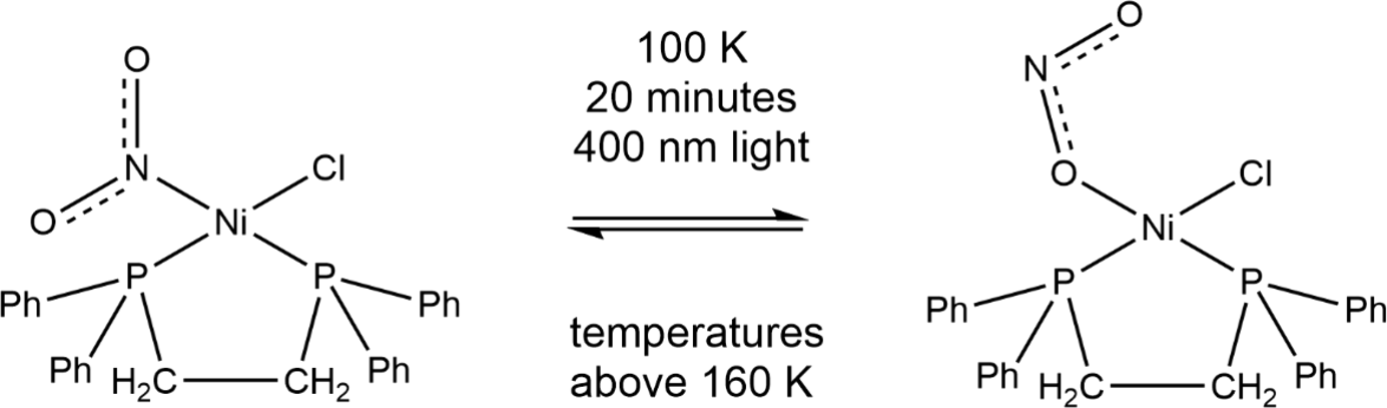
The reversible photoconversion of [Ni(dppe)(η^1^-NO_2_)Cl] into [Ni(dppe)(η^1^-ONO)Cl].

The first transition-metal nitro com­plex to display 100% efficient reversible linkage isomerism in the solid state under photoactivation was the square-planar nickel(II) com­plex [Ni(dppe)(η^1^-NO_2_)Cl] [dppe is 1,2-bis­(di­phenyl­phosphino)ethane], which was designed to contain the bulky dppe phos­phine ligand that would control the crystal packing and provide adequate space within the crystal lattice for the nitro→nitrito inter­conversion to occur (Warren *et al.*, 2009 ▸). The com­plex was studied by Raman spectroscopy and *photocrystallography*. Irradiation of a crystal with 400 nm light from an LED, below 160 K, resulted in the structural change from the ground state nitro com­plex to the *endo*-nitrito form (Fig. 3 ▸). At tem­per­a­tures above 160 K, the structure gradually reverted to the nitro form. This relaxation follows Arrhenius behaviour, with the lifetime of the *endo* form decreasing with increasing tem­per­a­ture, so that there is a specific tem­per­a­ture for a specific lifetime. This process was reversible without crystal degradation. Subsequent studies showed that the com­plexes *cis*-[Ni(dppe)(NO_2_)_2_] and *cis*-[Ni(dcpe)(NO_2_)_2_] both underwent com­plete reversible nitro→*endo*-nitrito con­version at 100 K, using 400 nm LED light, and that above 180 K the structures reverted to the nitro form (Warren *et al.*, 2014 ▸). The square-planar nickel(II) com­plex [Ni(3-{[2-(di­methyl­amino)­eth­yl]imino}-2-hy­droxy­imino-1-phenyl­propan-1-one)(NO_2_)], with the bulky imino ligand, which shows traces of the presence of the *exo*- and *endo*-nitrito (η^1^-ONO) forms in the ground state, undergoes 90% total conversion when irradiated with 405–530 nm LED light at tem­per­a­tures up to 200 K, with the *exo*-nitrito isomer being the dominant form (Potempa *et al.*, 2023 ▸). The relative populations of the *endo* and *exo* forms are tem­per­a­ture dependent.

**Figure 4:**
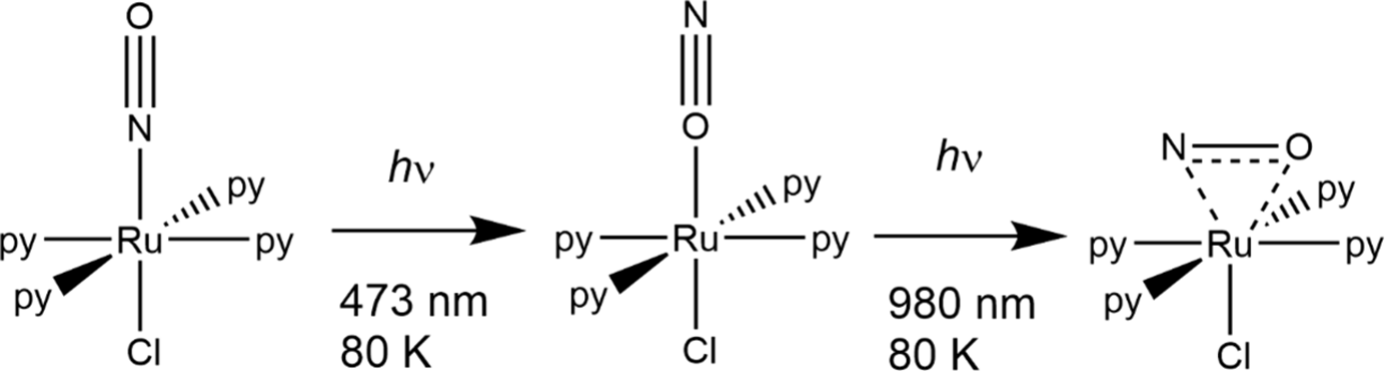
Photoactivated linkage isomerism in [RuCl(py)_4_(NO)](PF_6_)_2_·0.5H_2_O, showing the two *metastable* forms.

Schaniel and Woike obtained 92% conversion from the η^1^-NO-bound form to the η^1^-ON-bound form of the nitrosyl ligand in crystals of [RuCl(py)_4_(NO)](PF_6_)_2_·0.5H_2_O using laser light of 473 nm at 80 K, which could then be converted to 48% of the η^2^-NO form (Cormary *et al.*, 2009 ▸) (Fig. 4 ▸). The authors indicated that the conversion occurs *via* a metal-to-ligand charge-transfer process (MLCT) induced when the com­plex absorbs photons of the appropriate energy. This excitation must induce electron transfer between two orbitals to change the metal–nitrosyl bond, *e.g.* a *d*→π*(NO) transition. Also, for a *metastable* isomer to be generated, the potential energy surface for the excited state isomer must have a minimum point that directly overlaps with the maximum point of the ground state energy surface (Schaniel & Woike, 2009 ▸). The rate at which the excited state is populated must be greater than the rate of depopulation for the excited state to be identified. Since the ground and excited states will be in equilibrium, factors such as the tem­per­a­ture and illumination wavelength will influence the observed percentage conversion to the metastable state. These studies led to an extension of the work to include the spectroscopic and *photocrystallographic* investigation of a series of related com­plexes [Ru*X*(py)_4_(NO)](*Y*)_2_·*n*H_2_O (*X* = halide and *Y* = counter-anion) (Cormary *et al.*, 2012 ▸). It was found that the shorter the distance between the NO ligand and the counter-ion in the ground state structure, the higher the population of the *metastable* state after irradiation. This implies that the inter­molecular contacts in the *metastable* state are reduced, easing unfavourable packing constraints. Additionally, it was found that the lower the donating character of the ligand *trans* to the NO group in these octa­hedral com­plexes, the higher the photoconversion yield.

**Figure 5:**
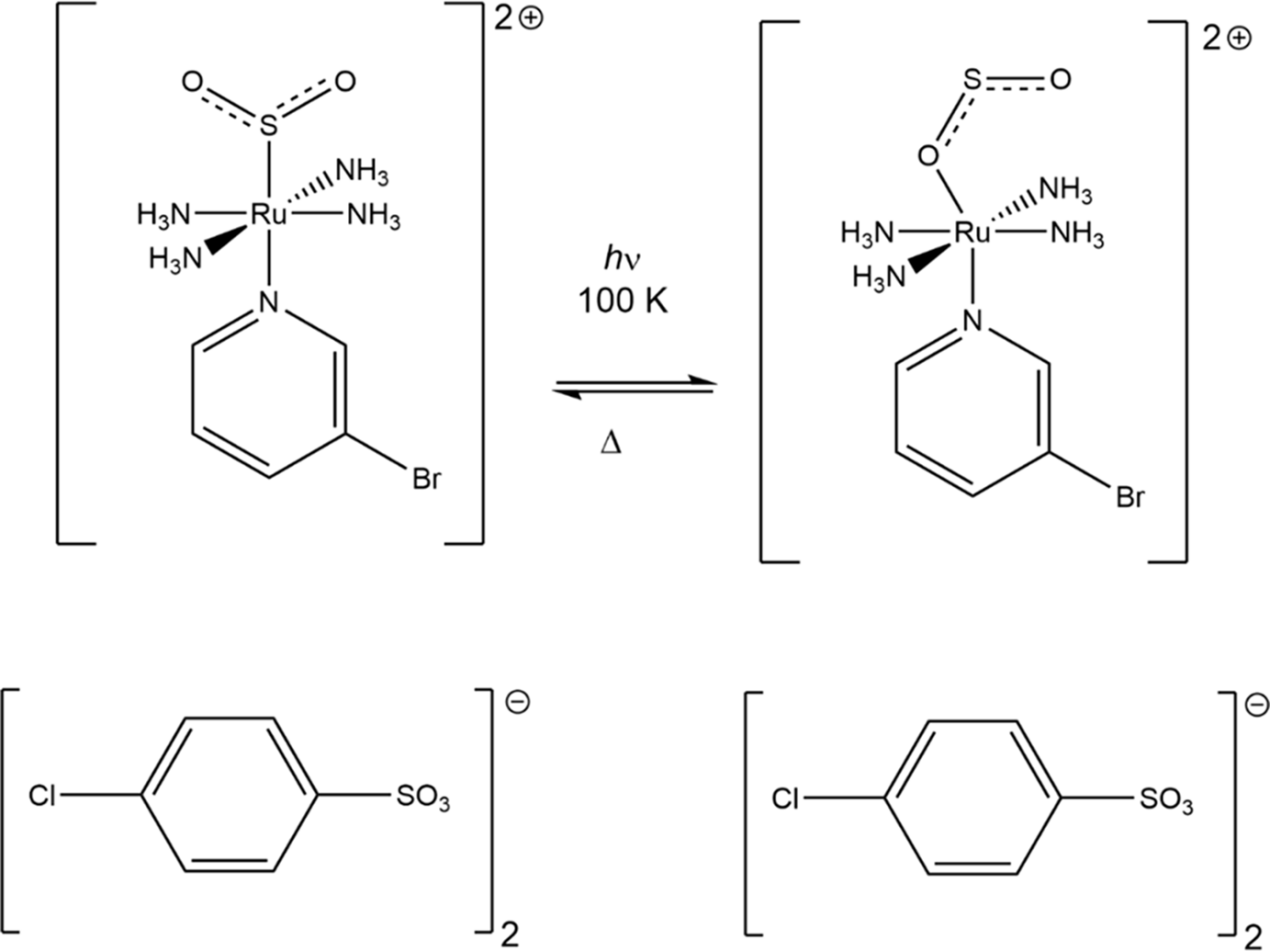
The η^1^-SO_2_ to η^1^-OSO photoisomerization in *trans*-[Ru(SO_2_)(NH_3_)_4_(3-bromo­pyridine)](tosyl­ate)_2_.

The *trans*-[Ru(SO_2_)(NH_3_)_4_(3-bromo­pyridine)](tosyl­ate)_2_ com­plex undergoes 100% conversion from the *S*-bound η^1^-SO_2_ isomer to the *O*-bound η^1^-OSO photoisomer, which is metastable at 100 K (Fig. 5 ▸) (Cole, Gosztola & Velazquez-Garcia, 2021 ▸). The *photocrystallographic* experiments have been supported by single-crystal optical absorption and Raman spectroscopies that confirm the metal-to-ligand charge-transfer nature of the process. Notably, single crystals of this material act as nano-optomechanical transducers that have the potential to be used in light-driven mol­ecular machinery, nanotechnology and quantum com­puting. The formation of the *metastable* η^1^-OSO isomer is the stimulus for the transduction, with the MLCT modulations relaying through the ruthenium cation, causing a knock-on effect in the tosyl­ate anion. The Br^δ−^ substituent on the cation inter­acts with the tosyl­ate ring on the anion through anion⋯π inter­actions and the ring rotates to accommodate this inter­action.

More recent experiments involving the nitro→nitrito inter­­conversion have resulted in the investigation of the pho­toisomerization of the square-planar Pd com­plex [Pd(Bu_4_dien)(η^1^-NO_2_)](BPh_4_) (Bu_4_dien = *N*,*N*,*N*′,*N*′-tetra­butyl­diethyl­enetri­amine and BPh_4_^−^ = tetra­phenyl­borate), that con­tains a bulky tridentate amine ligand and also has a bulky counter-ion, both of which reduce the efficiency of the crystal packing and leave more void space in the crystalline lattice. This com­plex undergoes 100% conversion to the *metastable endo*-nitrito-(η^1^-ONO) isomer in 15 minutes with irradiation by 400 nm LED light at a tem­per­a­ture of up to 240 K, while pseudo-steady-state *photocrystallographic* experiments, with continuous irradiation, showed that the excited state is retained at tem­per­a­tures up to 260 K (Hatcher, 2016 ▸). This example shows the promise of linkage isomerism occurring under near-ambient conditions. A subsequent crystallographic kinetic study on the nitrito to nitro decay in this mol­ecule confirms the Arrhenius-like decay behaviour, meaning that the excited state lifetimes can be tuned over several orders of magnitude through careful tem­per­a­ture control (Hatcher *et al.*, 2018 ▸). These results imply that it should be possible to ‘dial up’ a specific excited state lifetime, through choice of tem­per­a­ture, to suit the parameters of a specific time-resolved experimental set-up.

**Figure 6:**
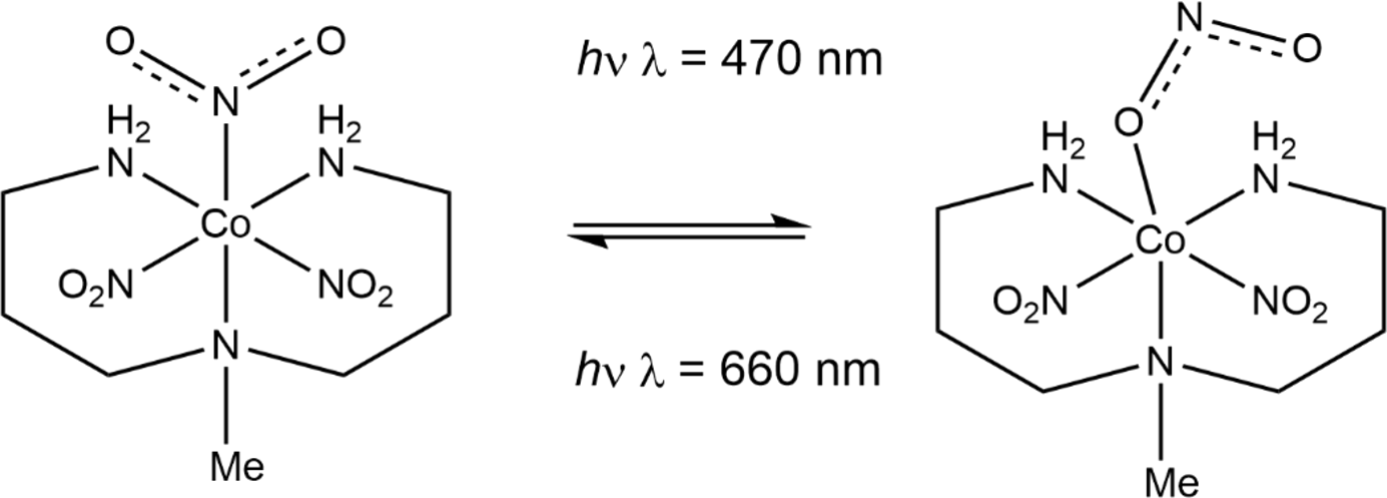
The light-induced transformation of [Co(Me-dpt)(NO_2_)_3_] between the nitro and nitrito forms.

Crystals of the *Pca*2_1_ polymorph of the cobalt(III) com­plex [Co(Me-dpt)(NO_2_)_3_] (Me-dpt = 3,3′-di­amino-*N*-methyl­pro­pane­di­amine) undergoes photoisomerization of one of the NO_2_ groups at room tem­per­a­ture, with a maximum nitro-to-nitrito conversion of 55%, when irradiated with 470 nm light, and the transformation is reversible when the crystal is irradiated with 660 nm light (Fig. 6 ▸) (Deresz *et al.*, 2022 ▸). Since there are three NO_2_ groups in the com­plex, and only one appears to undergo linkage isomerism, the reaction cavities for each NO_2_ group were calculated. Inter­estingly, the cavity for the NO_2_ ligand that does undergo isomerization is 7 and 5 Å^3^ larger than for the other two NO_2_ groups, respectively, with a volume of *ca* 30 Å^3^, which provides a *topotatic* explanation for the observed site selectivity. Solid-state IR and UV–Vis spectroscopy also monitored the reversibility of the isomerization.

The experiments discussed so far have been described as ‘steady state,’ where the lifetime of the excited state exceeds the length of the experiment, providing certain experimental conditions, such as tem­per­a­ture, are maintained and no further irradiation is required, or ‘pseudo-steady state’, where the excited state persists providing that the sample is con­tin­uously irradiated. However, obtaining the structures of species with shorter excited state lifetimes is possible using *stroboscopic* or *pump–probe photocrystallographic* techniques. In these experiments, short-duration pulses of light are used, which are synchronized with X-ray pulses that are synchronized to arrive at the crystalline sample in a specific time sequence (Fullagar *et al.*, 2000 ▸), so that the X-ray data is only measured when the crystal is photoactivated. Usually, a laser is used that generates nanosecond or picosecond pulses, although pulsed LED sources have been used successfully on the millisecond to microsecond timescale (Hatcher *et al.*, 2022 ▸). The laser or LED light acts as the *pump*, while the X-ray pulses function as the *probe*.

**Figure 7:**
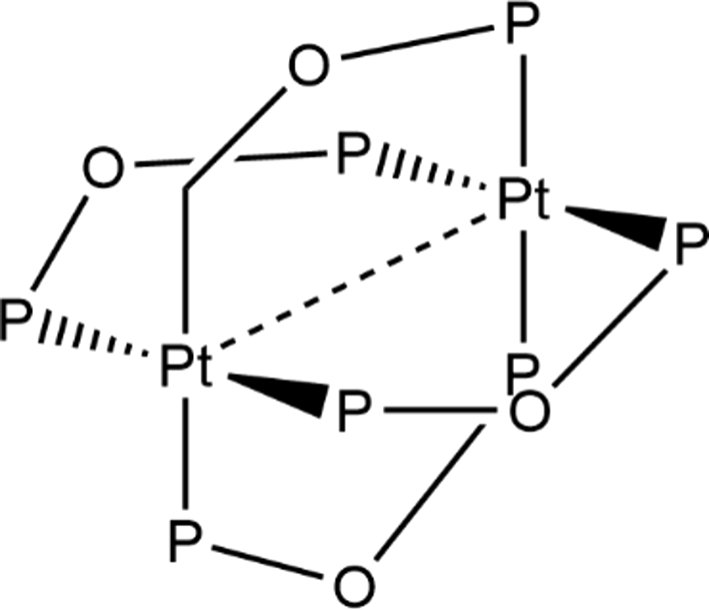
The core of the [Pt_2_(pop)_4_]^4−^ tetra­anion. The Pt⋯Pt separation reduces by 0.28 (9) Å upon photoactivation.

Coppens was among the first mol­ecular crystallographers to use the higher intensity X-ray flux available at synchrotrons to conduct *photocrystallographic* experiments on mol­ecules with microsecond lifetimes. In 2002, he conducted pump–probe experiments on salts of the tetra­anion [Pt_2_(pop)_4_]^4−^ (pop = [H_2_P_2_O_5_]^2−^) (Fig. 7 ▸) using monochromatic X-ray radiation. In the case of the tetra­ethyl­ammonium salt, the anion showed structural distortions when photoactivated by 355 nm laser light, producing a triplet excited state with a microsecond lifetime (Kim *et al.*, 2002 ▸). The anion showed a 2% conversion to the excited state, at 17 K, using 33 µs wide light pulses from a Nd/YAG laser with a repetition rate of 5100 Hz. The refinement of the excited state focused on changes in the Pt⋯Pt separation, while the remainder of the structure was treated as a rigid group. The Pt⋯Pt distance was found to shorten by 0.28 (9) Å, with an accom­panying rotation of 3° about the Pt⋯Pt vector. In a subsequent diffraction study on [(*n*-Bu_4_N)_2_H_2_][Pt_2_(pop)_4_], Ohashi showed a decrease in the Pt⋯Pt distance of 0.23 Å upon photoactivation (Ozawa *et al.*, 2003 ▸).

The dimetallic com­plex [Rh_2_(dimen)_4_](PF_6_)_2_·MeCN (dimen = 1,8-diiso­cyano­methane) also shows a reduction of 0.86 Å in the Rh—Rh bond and a bond rotation of 13° upon photoactivation with 335 nm laser light at 23 K (Coppens *et al.*, 2004 ▸). The maximum level of excitation reached was 2.5%, with an excited state lifetime of 11.7 µs. Two copper com­plexes have also been studied using pump–probe methods with monochromatic radiation, *i.e.* [Cu_3_{3,5-(CF_3_)pyrazolate}_3_] (Vorontsov *et al.*, 2005 ▸) and [Cu(dmp)(dppe)](PF_6_) (dmp = 2,9-dimethyl-1,10-phenanthroline) (Vorontsov *et al.*, 2009 ▸). In the former case, photoactivation with 355 nm laser light at 17 K causes a rearrangement of adjacent Cu_3_ triangular units into pairs such that one interplanar Cu⋯Cu inter­molecular distance is reduced by 0.65 Å, and the separation to the next pair of Cu_3_ triangles is increased by 0.30 Å. In [Cu(dmp)(dppe)](PF_6_), there are two independent mol­ecules in the asymmetric crystallographic unit. Upon excitation, one of the pseudo-tetra­hedral mol­ecules flattens out more than the other, with a concomitant increase in the Cu—P distances. This difference is attributed to the slightly different crystalline environments of the two mol­ecules. The population of the excited state was estimated to be in the range 7–10%.

An alternative approach to using monochromatic X-ray radiation in time-resolved crystallographic experiments is to use Laue diffraction techniques while pumping the crystalline material with laser or LED-generated light. In Laue experiments, a ‘white beam’ including a range of X-ray wavelengths is used, which provides a much wider energy range of X-ray photons (Moffat, 2019 ▸). This technique vastly increases the number of X-ray reflections measured per diffraction frame recorded, thus speeding up the experiment, reducing the risk of crystal damage and potentially allowing systems with shorter excited state lifetimes to be probed. The com­plication is that each diffraction spot recorded must be matched with the X-ray wavelength that generated it.

In 2011, Coppens first used Laue diffraction methods to attempt a ‘single-shot’ experiment on the mol­ecular dirhodium com­plex [Rh_2_(μ-pnp)_2_(pnp)_2_](BPh_4_) [pnp = *N*,*N*-bis­(di­meth­oxy­phosphan­yl)methanamine] (Benedict *et al.*, 2011 ▸; Makal *et al.*, 2011 ▸). The polychromatic X-ray source was used at the 14-ID BioCARS Station at the APS. A small single crystal of the sample was cooled to 225 K and irradiated with a 35 ps-wide laser pulse with a wavelength of 337 nm and then exposed to a single 100 ps X-ray pulse after a 100 ps delay. This excitation populated the triplet state and resulted in the transient shortening of the Rh—Rh bond distance, similar to that observed previously for [Rh_2_(dimen)_4_](PF_6_)_2_·MeCN when monochromatic X-ray radiation had been used (Coppens *et al.*, 2004 ▸).

A copper(I) com­plex, [Cu(1,10-phenanthroline)(PPh_3_)_2_](BPh_4_), has also been studied using Laue techniques (Makal *et al.*, 2012 ▸), and the results com­pared with those obtained for the similar com­plex [Cu(dmp)(dppe)](PF_6_), whose excited state structure had been obtained using monochromatic X-ray radiation in a time-resolved crystallographic experiment (Vorontsov *et al.*, 2009 ▸). As in the previous case, [Cu(1,10-phenanthroline)(PPh_3_)_2_](BPh_4_) crystallizes with two independent cations in the asymmetric unit, and from analysis of the inter­molecular contacts in the ground state structure, one of the cations exhibits a more sterically constrained crystalline environment than the other. Photoexcitation with 390 nm light at 90 K induces an MLCT transition, but the two independent mol­ecules undergo different structural changes. X-ray data were recorded using the single-pulse Laue method (Kalinowski *et al.*, 2012 ▸). The analysis showed that the less sterically crowded cation exhibited a considerable distortion, but no significant changes in the more constrained cation.

**Figure 8:**
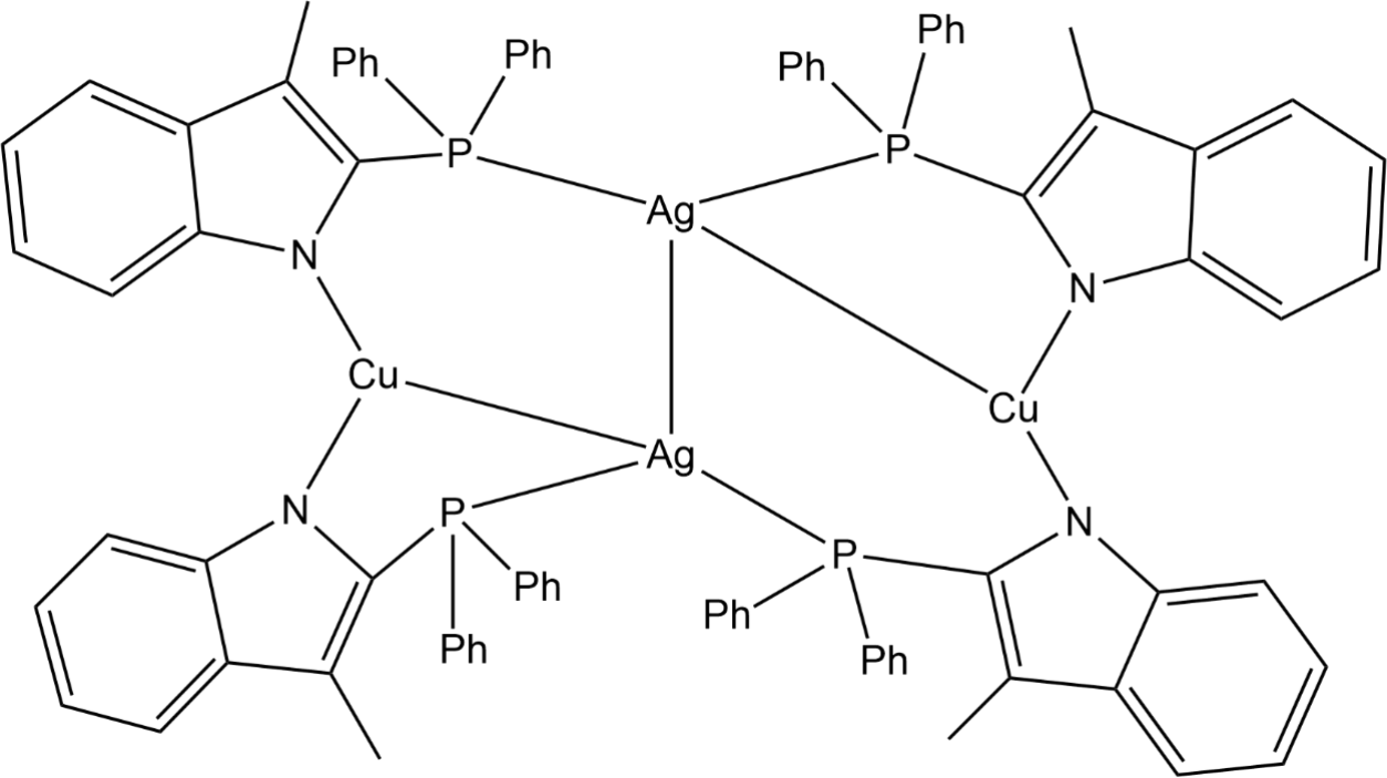
The mol­ecular structure of [Ag_2_Cu_2_(2-di­phenyl­phosphino-3-methyl­in­dole)_4_].

A ‘pink-beam’ Laue diffraction study, in which a restricted range of X-ray wavelengths are employed, has been carried out on a tetra­nuclear Cu^I^–Ag^I^ com­plex, [Ag_2_Cu_2_(2-di­phenyl­phosphino-3-methyl­indole)_4_] (Fig. 8 ▸) (Jarzembska *et al.*, 2014 ▸). The triplet excited state, which has a 1 µs lifetime at 90 K, is activated by 390 nm light pulses and probed with 80 ps resolution. The ‘zigzag’ arrangement of the two Cu and two Ag centres shows a reduction in the Ag⋯Cu distance of 0.59 (3) Å and a shortening of the Ag⋯Ag distance by 0.38 (3) Å, suggesting a strengthening of the *d*^10^⋯*d*^10^ inter­actions. An accom­panying quantum chemical study confirms that the strengthening of the Ag⋯Ag inter­action is the result of ligand-to-metal charge transfer (LMCT).

**Figure 9:**
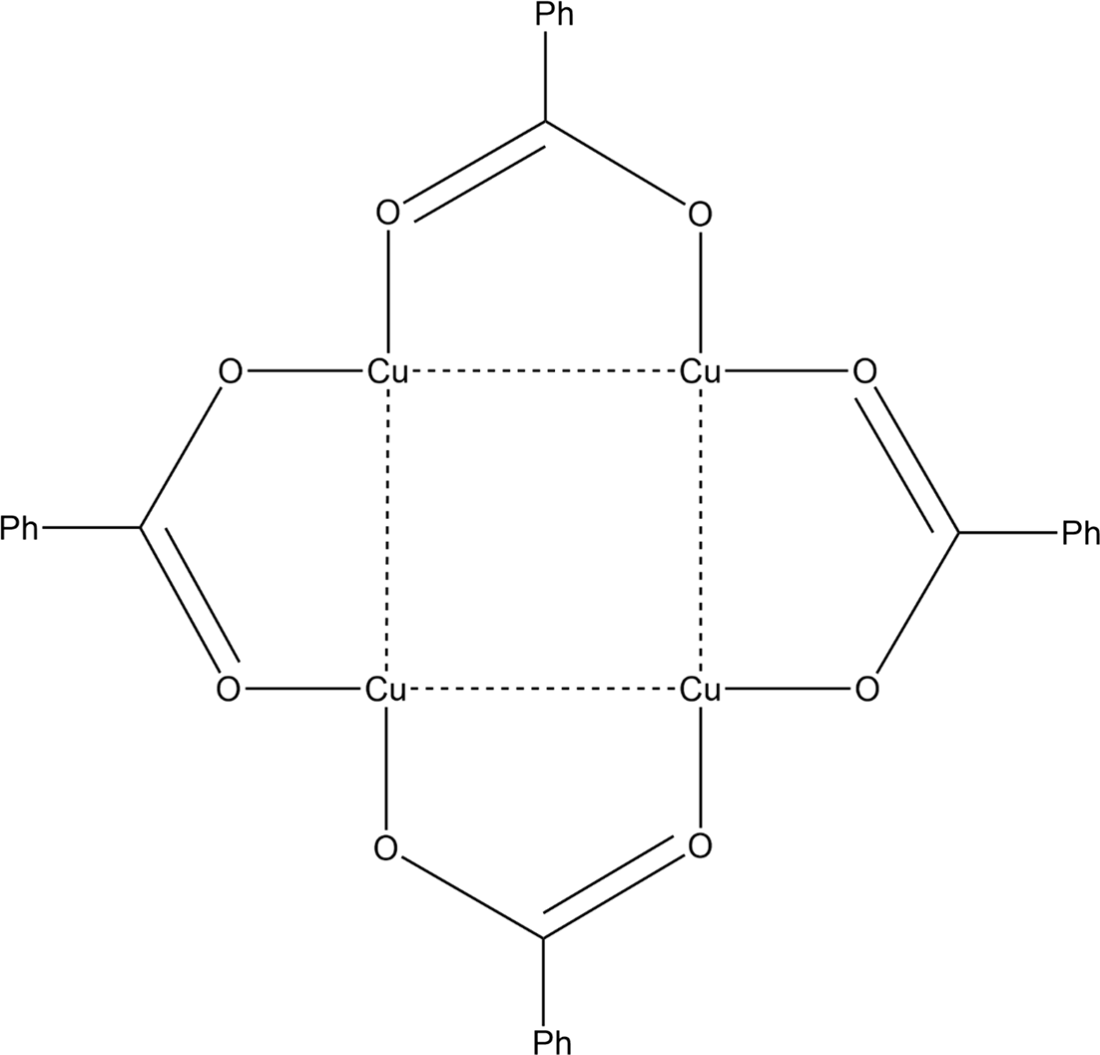
The mol­ecular structure of [Cu_4_(PhCO_2_)_4_].

The copper(I) benzoate com­plex [Cu_4_(PhCO_2_)_4_] (Fig. 9 ▸) has been studied using time-resolved Laue diffraction techniques at 90 and 225 K, using 355 and 360 nm light, and this shows an expected Cu⋯Cu contraction in the solid state (Jarzembska *et al.*, 2019 ▸). As in previous examples, the asym­metric unit contains two independent mol­ecules, each displaying slightly different distortions. The com­plex shows luminescent thermochromism at 90 K, and the low-energy triplet state has been assigned to the Cu_4_ core. The emission from this state matches the red band at 660–715 nm.

## Timescales

**Figure 10:**
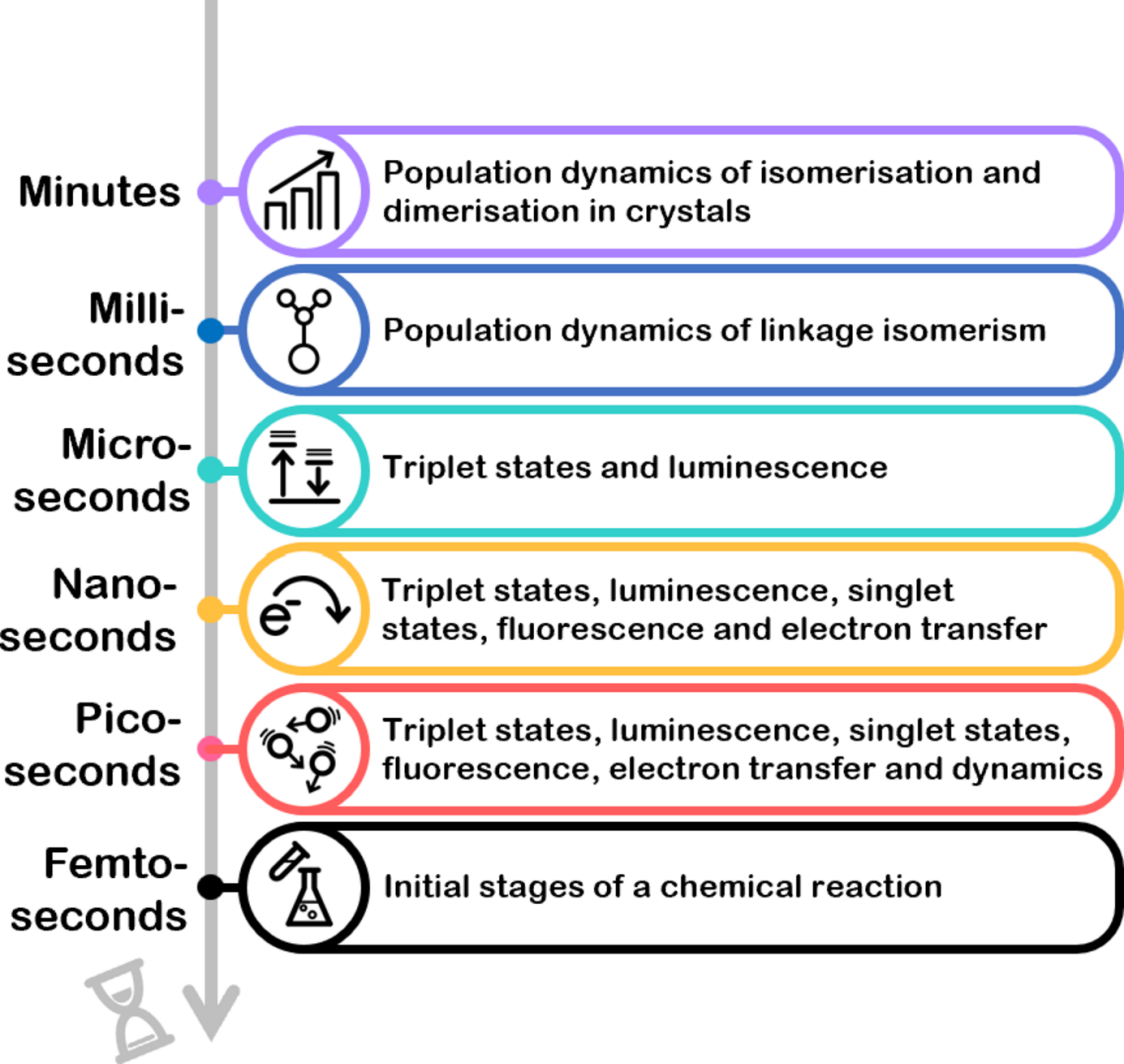
The timescales of dynamic processes that occur in chemistry.

Photochemical processes can occur on timescales ranging from femtoseconds to years, and distinct aspects of a reaction occur on quite different timescales (Fig. 10 ▸). Some processes are irreversible, and the reaction pathway occurs only in the forward direction, although, depending on the reaction conditions, it may take periods of hours or days to reach 100% conversion to the reaction product, such as in the case of some [2 + 2] cyclo­addition reactions (Allen *et al.*, 2005 ▸; Mahon *et al.*, 2008 ▸). Other processes are reversible, and it is for these systems that photocrystallography can be used to monitor the forward and backward reactions, covering a whole range of timescales. The timescale of a particular process is susceptible to a variety of external factors, perhaps most commonly changes in tem­per­a­ture, so, for example, an isomer may be metastable under irradiation at one tem­per­a­ture, but at a higher tem­per­a­ture, it may have a lifetime of microseconds. Generally, electron-transfer processes within a mol­ecule can occur in picoseconds, whereas the population dynamics of excited state formation throughout a crystal can vary over timescales of several minutes. If the processes are to be monitored or snapshots taken along a reaction pathway, thought needs to be given to the methodology to be used to pick out the feature of the reaction that is of inter­est. When using crystallographic techniques, the shorter the timescale of the process, the more com­plicated and challenging the crystallographic experiment required (Pillet, 2018 ▸; Raithby, 2020 ▸).

Given the caveats mentioned above, Fig. 10 ▸ contains a summary of common light-induced processes and the timescales on which they may occur.

### ‘Steady-state’ experiments

**Figure 11:**
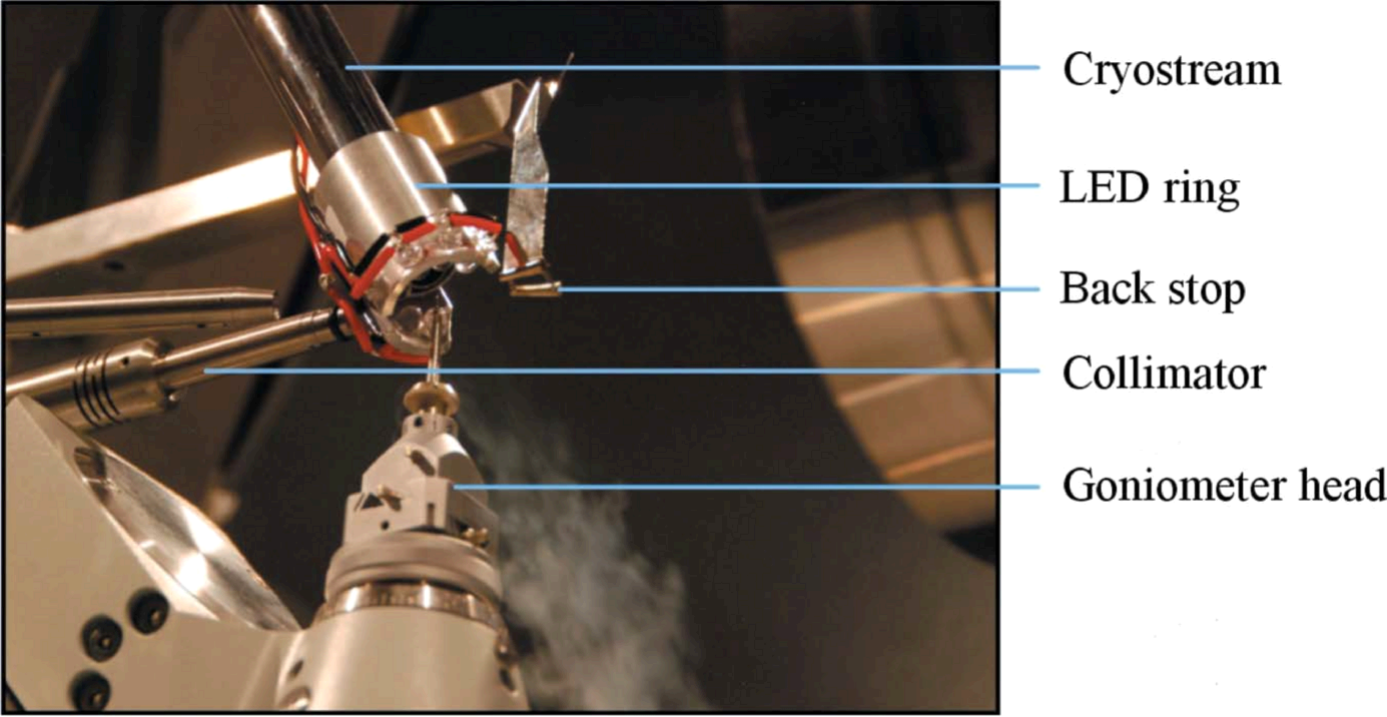
An Oxford Diffraction Gemini A Ultra diffractometer equipped with an Oxford Cryostream crystal-cooling device and a ring of LEDs to illuminate the crystal. [Reproduced from Brayshaw *et al.* (2010 ▸) with permission from the Inter­national Union of Crystallography.]

In *photocrystallographic* experiments where the excited state or metastable state lifetime of the system is measured in hours and is longer than the duration of the diffraction experiment, conventional single-crystal X-ray diffraction techniques are used to determine the structures of the ground and excited states. They are described as ‘steady-state’ experiments. A laboratory-based diffractometer with an area detector can be used, although using a diffractometer at a synchrotron facility will substanti­ally speed up the experiments, particularly if multiple data sets are collected over a range of tem­per­a­tures and under different illumination conditions. The flux of the synchrotron X-ray source is several orders of magnitude higher than that from a conventional laboratory instrument. The diffractometer should be equipped with a crystal-cooling apparatus capable of maintaining tem­per­a­tures between 100 and 500 K, and the ability to reach down to tem­per­a­tures of a few degrees K is necessary for some experiments. Most importantly, for *photocrystallographic* experiments, the diffractometer must be equipped with a light source to illuminate the crystal. The light source may be a lamp, a laser or a ring of LEDs, depending on the requirements of the study to be conducted (Fig. 11 ▸). Attempting to irradiate the crystal during a diffraction experiment when the diffractometer is moving position to record data can be challenging and several ingenious set-ups have been devised to achieve this using either a laser (Thompson *et al.*, 2004 ▸; Kamiński *et al.*, 2016 ▸) or LEDs (Brayshaw *et al.*, 2010 ▸).

In a typical single-crystal *photocrystallographic* experiment to study the structure of a com­plex that exhibits a long-lived metastable state under light irradiation using the set-up described above, the crystal is first cooled in the dark to a low tem­per­a­ture to obtain the ground state structure with minimum risk of contamination from the metastable state. A conventional high-resolution data set is then collected in the dark at the low tem­per­a­ture selected. The crystal is then illuminated for a set period, usually ranging from minutes to hours, depending on the knowledge obtained from preliminary spectroscopic and photochemical studies. The metastable state is kinetically trapped at low tem­per­a­tures, and a significant steady-state population builds during irradiation. Depending on the system, the selected tem­per­a­ture is usually from a few degrees K to around 150 K. A second conventional high-resolution data set is then collected to obtain the metastable state structure. In fortuitous and rare cases, com­plete conversion from the ground state structure to 100% of the metastable structure has occurred. More commonly, the resultant structure contains a mixture of ground and metastable states, resulting in a disordered picture. To resolve this disorder, the crystal is usually irradiated for a further period, and then subsequent data are collected. This cycle is repeated until the maximum photostationary state population is reached. Once the maximum population has been identified, the crystal is often maintained in the dark, and a series of data collections at different tem­per­a­tures are conducted to establish the tem­per­a­ture range over which the metastable state is maintained and at what rate it will decay at a given tem­per­a­ture. The structures of many of the metastable nitrosyl, nitrite and sulfur dioxide com­plexes have been determined using this methodology (Bowes *et al.*, 2006 ▸; Hatcher *et al.*, 2019 ▸).

**Figure 12:**
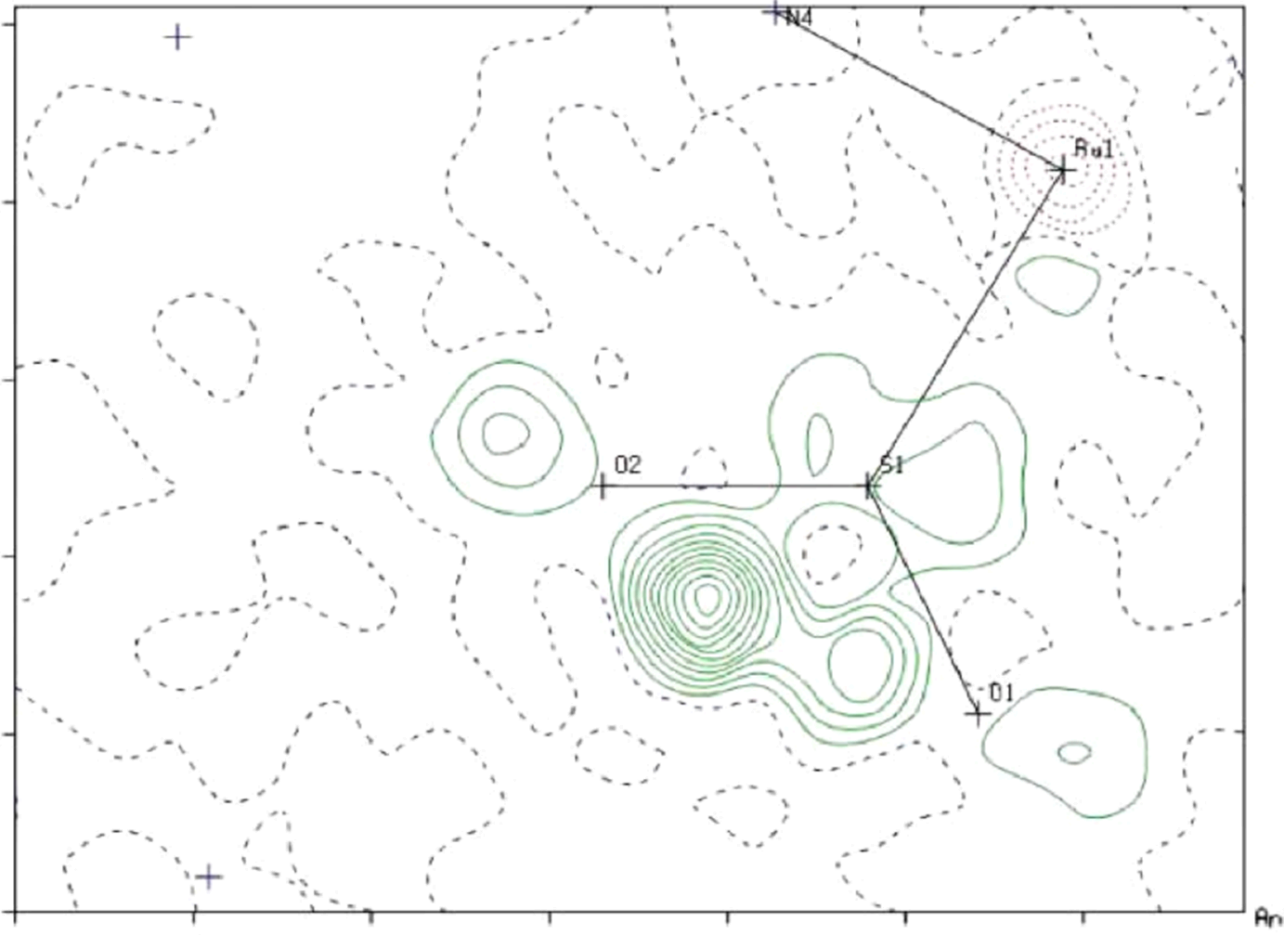
The electron-density difference map drawn through the ground state Ru1/S1/O1/O2 plane calculated using the rigid-body ground state structure and the X-ray data set recorded after photoactivation. The highest residual electron density in the map (green) shows the positions of the two disordered com­ponents of the (η^1^-SO_2_) linkage isomer. [Reproduced from Bowes *et al.* (2006 ▸) with permission from the Royal Society of Chemistry.]

Processing of the ground and excited state data sets and com­paring them can be challenging, particularly if 100% conversion to the excited state structure does not occur. There will be small changes in the unit-cell dimensions between the clean ground state structure and the structures that contain com­ponents of the ground and excited states, and indeed of the 100% excited state structure if photoactivation occurs within the single crystal. In order to accommodate these changes, the coordinates of the ground state crystal need to be normalized relative to the changes associated with the unit-cell dimensions of the photoactivated data set. The resulting coordinates can then be used to represent the ground state com­ponent of the model as a fixed rigid body in the refinement of the photoactived data. Examination of the photoelectron density difference maps can then be used to show the disorder com­ponents of the excited state structures within the crystal, which can be refined in the disorder model. Once the disorder com­ponents have settled, the coordinates for all the other atoms not involved in the photoexcitation process can be released from their rigid-body constraints, and the atomic positions and displacement parameters are allowed to refine freely, all with occupancies set to unity (Bowes *et al.*, 2006 ▸). An example of a photoelectron density difference map for the structure of the cation in the [Ru(NH_3_)_4_(H_2_O)(η^1^-SO_2_)](MeC_6_H_4_SO_3_)_2_ salt is shown in Fig. 12 ▸. Upon excitation with a tungsten lamp at 13 K, for 75 minutes, the (η^1^-SO_2_) isomer was formed with a population of *ca* 35% (disordered over two orientations). In the difference map, the two orientations of the (η^1^-SO_2_) linkage isomer are shown in green (Bowes *et al.*, 2006 ▸).

In some cases, it may not be possible to obtain a stable refinement of the mixed ground state and excited state dis­order model, and then constraints or restraints on some bond parameters may have to be retained in the final refinement cycles. The values to be used in any constraints or restraints should be chemically sensible and may be taken from either a fully-ordered structure of the same material (*e.g.* a ‘clean’ 100% ground state or 100% excited state structure, where available), or else from another suitable experimental source (*e.g.* by identifying the average values for that bond type though analysis of similar crystal structures in the Cambridge Structural Database; Groom *et al.*, 2016 ▸). Under those circumstances, while it is possible to identify the presence of different isomers from changes in the positions of the atoms involved, it may not be possible to comment qu­anti­tatively on changes in the bond parameters of the isomers present, and certainly not if the bond lengths or angles are constrained in the refinement. This is particularly true if the level of excitation to produce the excited state isomer is not high. For example, in a detailed study of the [Ru(py)_4_Cl(NO)](PF_6_)_2_ salt, Schaniel and Woike showed that for nitrosyl isomers with less than 50% excited state populations, only a qualitative assessment of the bonding could be given (Cor­mary *et al.*, 2009 ▸). In cases where it may be possible to com­pare the bond parameters in different isomers, the estimated standard deviations should be analysed carefully before any qualitative com­parisons can be made.

### ‘Pseudo-steady-state’ experiments

As mentioned in the *Introduction*, an alternative to the ‘steady-state’ experiment is a ‘pseudo-steady-state’ experiment. In a ‘steady-state’ experiment, the excitation and decay processes can be considered to be independent. In a ‘pseudo-steady-state’ experiment, the crystal is continuously irradiated throughout the data collection, the excitation and decay are com­petitive, and an equilibrium between the two processes will be reached.

**Figure 13:**
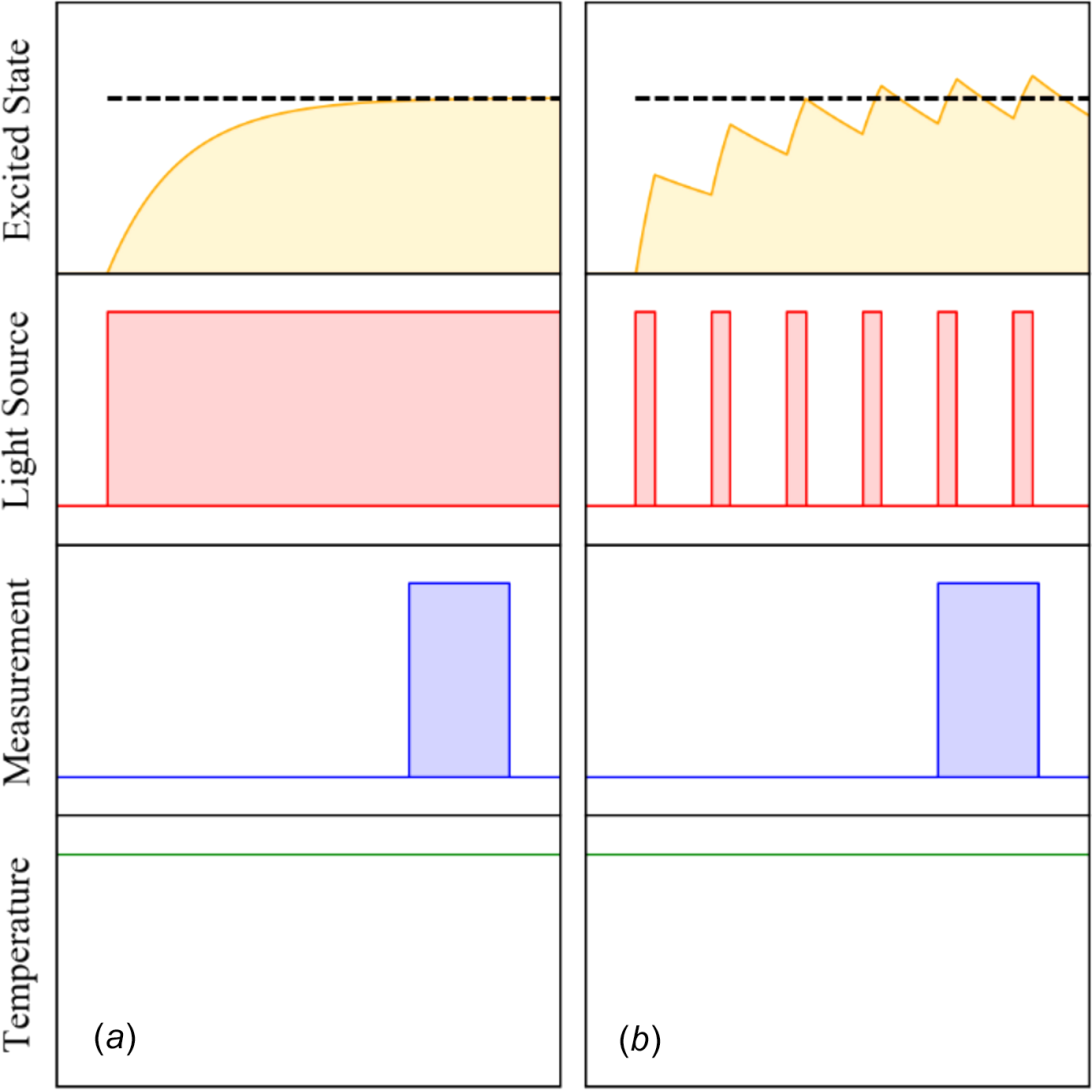
Schematic of a typical (*a*) ‘steady state’ and a (*b*) ‘pseudo-steady-state’ experiment. In part (*a*), the sample is illuminated continuously, and the excited state population builds to a steady-state value. This equilibrium population, marked by a dashed black line, is then measured after a pre-determined equilibration time. In part (*b*), the sample is illuminated with a pulsed laser or LED, resulting in the excited state population oscillating about an equilibrium value. If the measurement time is slower than the pulse frequency, the average population, again denoted by the dashed black line, is measured. [Reproduced from Hatcher *et al.* (2020 ▸) with permission.]

There are slight differences in the methodology of the experiment depending on whether a continuous or a pulsed radiation source is used. Both lasers and LEDs can either provide a constant stream of illumination, or the illumination can be pulsed with short pulses of illumination with precise time inter­vals between them. The two situations are shown in Fig. 13 ▸ (Hatcher *et al.*, 2020 ▸).

The ‘pseudo-steady-state’ method has been used to advantage in several instances, highlighting features of the structural dynamics of the linkage isomerization process that are not apparent when using ‘steady-state’ methods. For example, using a ‘steady-state’ methodology, single crystals of [Ni(Et_4_dien)(η^2^-O,ON)(η^1^-NO_2_)] (Et_4_dien = *N*,*N*,*N*′,*N*′-tetra­ethyl­diethylenetri­amine) can be irradiated with 500 nm light at tem­per­a­tures below 150 K to generate the *endo*-nitrito isomer [Ni(Et_4_dien)(η^2^-O,ON)(η^1^-ONO)] in 100% yield. How­ever, when the crystal is continuously pumped with the 500 nm light source during the data collection, a previously unobserved *exo*-nitrito (η^1^-ONO) linkage isomer is detected (Hatcher, Christensen *et al.*, 2014 ▸). The use of ‘pseudo-steady-state’ methods is also helpful in studying the decay of metastable isomers above their metastable limit. Since the decay rate generally increases with tem­per­a­ture while the excitation rate remains relatively constant, the ‘steady-state’ excited state population falls from its maximum as the tem­per­a­ture increases. For crystals of the salt [Pd(Bu_4_dien)(η^1^-NO_2_)](BPh_4_), under irradiation, the decay of the excited state *endo*-(η^1^-ONO) isomer to the ground state (η^1^-NO_2_) becomes com­petitive at around 230 K, and between 230 and 290 K the observable excited state population drops to zero (Hatcher *et al.*, 2018 ▸) as the decay process swamps the excitation.

### Pump–probe experiments

As the lifetimes of the excited state species become shorter, more com­plicated methodologies are required to monitor the time evolution of the excited state structure during the X-ray experiment. Ideally, com­plete X-ray data sets could be collected within the duration of one excitation pulse or, more realistically, an excited state equilibrium needs to be set up with a repetitive pump–probe methodology, where the crystalline species is repeatedly pumped up into an excited state, and the resultant structure is probed by an X-ray pulse only when it is excited. Hence, these techniques are described as pump–probe methods or stroboscopic methods. These methods are suitable for studying species with lifetimes of milliseconds and shorter. The technique is called Time-Resolved Single-Crystal X-ray Diffraction (TR-SCXRD), which is in part to differentiate it from the studies of metastable and long lifetime systems (Raithby, 2020 ▸).

For these experiments, the light pump pulse has to be synchronized with the X-ray probe pulse so that the X-ray pulse records the diffraction pattern when the crystal is in the excited state and not when the excited state has deca­yed back to the ground state. In the early pump–probe experiments, the X-ray source was inter­rupted by a mechanical chopper (Fullagar *et al.*, 2000 ▸; Husheer *et al.*, 2010 ▸), so that the X-rays were blocked when the crystal had deca­yed to the ground state. More recently, mechanical choppers have been replaced by electronically time-gated pixel detectors (Casaretto *et al.*, 2017 ▸).

**Figure 14:**
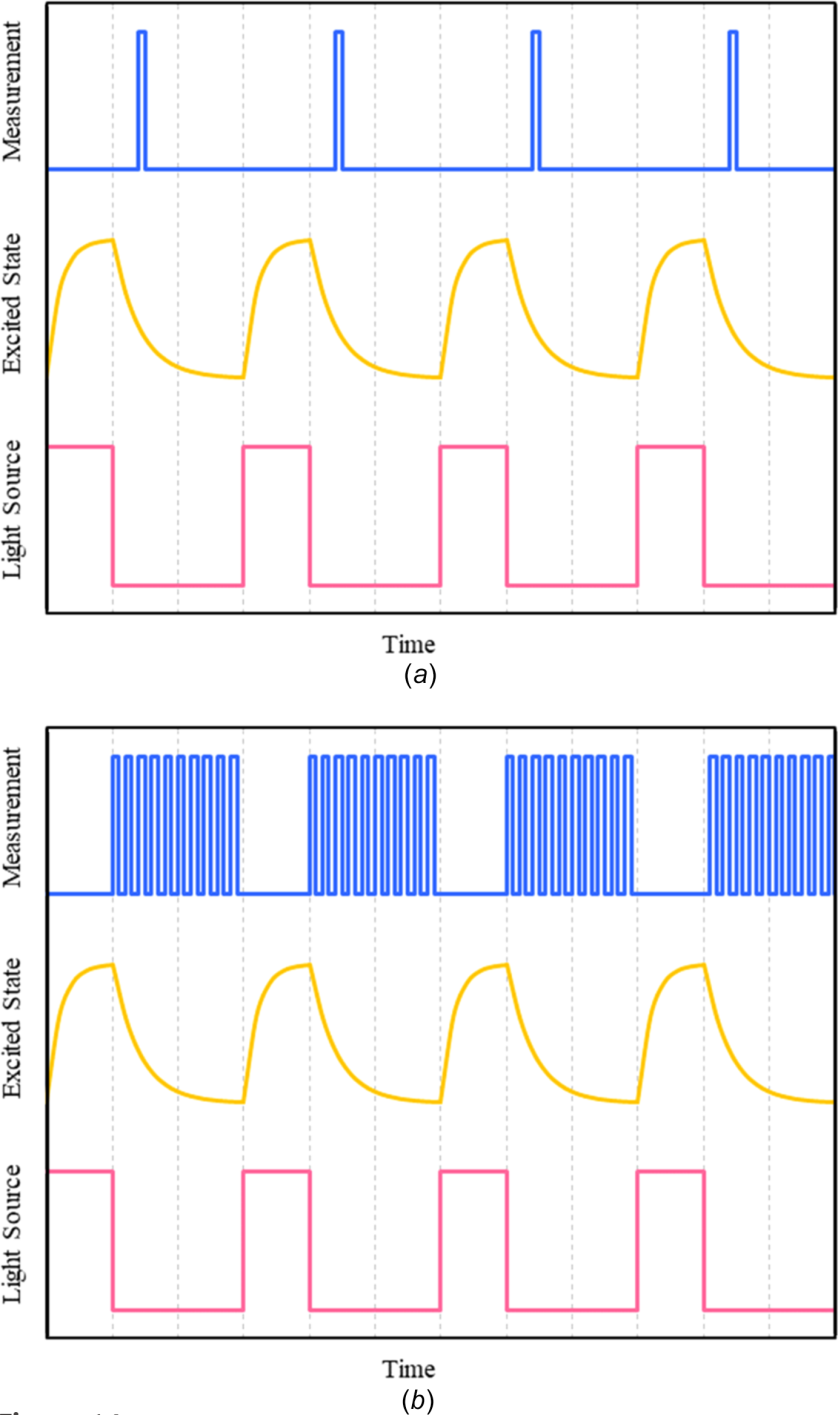
(*a*) In a pump–probe experiment, a single X-ray probe pulse is timed to measure the excited state population at a specific time delay Δ*t* after excitation by the pump light source. The com­plete experiment is repeated for each Δ*t* to be measured. (*b*) In the pump–multiprobe method, a series of probe pulses are generated after each pulse to measure multiple time delays in a single experiment. [Reproduced from Hatcher *et al.* (2020 ▸) with permission.]

Pump–probe experiments are most commonly run at synchrotron sources, where the probe synchrotron beam has a time structure and is essentially pulsed. However, depending on the lifetime of the species to be investigated, mechanical choppers or time-gated detectors are often used. The short-duration light pulses and the monochromatic X-ray pulses are synchronized to arrive at the crystal in a specific time sequence (Fullagar *et al.*, 2000 ▸). A repeating sequence is set up in which the crystal is excited by the light pulse at time *t* = 0 and is allowed to decay to the ground state before the next pulse arrives. The X-ray probe pulse is synchronized with the light pulse so that the diffraction pattern is collected after a fixed time delay Δ*t*. The pump pulse generates an excited state population, and the probe pulse measures the population over a brief time consistent with the time resolution of the experiment. The pump–probe sequence for a typical TR-SCXRD experiment is illustrated in Fig. 14 ▸(*a*). The technique has been used successfully by several research groups (Deresz *et al.*, 2021 ▸), most notably by Coppens (2017 ▸) to determine the photoactivated structures of mol­ecular com­plexes with lifetimes in the microsecond range. However, many pump–probe cycles are required per data collection frame to build up a strong diffraction image, and many frames are necessary to obtain a com­plete X-ray data set so that a single experiment may take many hours. Additional drawbacks to the method are that the repeated pumping and probing with high-intensity beams causes crystal damage, and there may also be heating effects that are detrimental to the crystal.

### Pump–multiprobe experiments

Pump–multiprobe techniques have been designed to overcome some of these disadvantages and speed up the X-ray data collection. The ability to perform pump–multiprobe experiments has been enhanced by the development of electronically time-gated pixel detectors with fast readout times that make it possible to record the output from multiple probe pulses at different time delays, Δ*t*, following a single pump pulse, with the total signal from each probe sequence being recorded as a single measurement, as illustrated in Fig. 14 ▸(*b*). With time-gated or pixel detectors, the photon-counting statistics determine the quality of the X-ray data recorded, and there is no dark current or read-out noise to inter­fere with the signal recorded as occurs with conventional CCD detectors. The concept of pump–multiprobe experiments was developed initially by macromolecular crystallographers using the Hada­mard transformation (Yorke *et al.*, 2014 ▸). The sensitivity of the experiment is defined by the number of photons in the whole probe sequence, with the time resolution being defined by the com­plete length of the probe sequence divided by the number of pulses. Application of the method means that the time resolution is no longer limited to X-ray flux by summing the time points across the probe sequence. The signal-to-noise ratio is also improved because of the increased number of photons recorded during the experiment.

Over the last five years, time-gated detectors have been supplied with new laboratory-based diffractometers. So, with appropriate laser or LED equipment, the synchronization of light and X-ray pulses is possible across a wide range of timescales by electronically gating the detector so that the X-ray diffraction pattern is only measured when the crystal is activated (Casaretto *et al.*, 2017 ▸). Building on these advances, a bank of pulsed LEDs has been combined with a time-gated Timepix 3 detector, at the Diamond Light Source, to conduct pump–multiprobe analysis of the linkage isomerism in [Pd(Bu_4_dien)(η^1^-NO_2_)](BPh_4_), at close to room tem­per­a­ture, and this produced a time-resolved ‘mol­ecular movie’ with a 400 millisecond time resolution (Hatcher *et al.*, 2022 ▸).

### Laue diffraction experiments

As mentioned earlier in the discussion, an alternative approach to using monochromatic X-ray radiation for TR-SCXRD experiments is to employ polychromatic or Laue X-ray radiation (Schmøkel *et al.*, 2010 ▸; Makal *et al.*, 2011 ▸). With the broader range of energies used, the flux of the X-ray beam is higher than from a monochromatic source, meaning that time-resolved X-ray data can be collected more quickly. Generally, a pink Laue beam is used rather than a white beam. Here ‘pink’ means a beam containing a small range of wavelengths rather than a white beam with all wavelengths. Because of the higher X-ray intensity, the number of pump–probe cycles in a pump–probe data collection can be significantly reduced (Coppens, Vorontsov *et al.*, 2005 ▸) and, as a consequence, crystal decay and crystal heating problems may be reduced. However, because the diffraction pattern ob­tained contains diffraction spots with different wavelengths, it is more difficult to process and inter­pret the data. A wavelength dependence correction has to be applied. Additionally, because mol­ecular crystal structures have relatively small unit cells, there are relatively few diffraction spots on any given frame of data, and the scaling of intensities can be challenging. To meet this challenge, Coppens developed the RATIO method (Coppens *et al.*, 2009 ▸), in which the intensity difference for each reflection is identified by using *on*/*off* ratios as the observables in the refinement of the excited state structure using the *LASER* software (Vorontsov *et al.*, 2010 ▸). For this method to be successful, the laser-*on* and laser-*off* intensities for each reflection must be measured immediately after one another. This approach eliminates the effect of any slow crystal deterioration, and scaling is not required since the paired data frames are collected under the same conditions.

Once the Laue data have been collected and processed, Fourier difference maps can be com­puted to analyse the structural changes that occur upon excitation of the crystal (Fournier & Coppens, 2014 ▸). As the RATIO method has been used in data processing, the com­puted photodifference map is based on the difference between the observed laser-*on* and laser-*off* structure factors. The method has been shown to be useful in dynamic spin-state photoswitching crystallographic studies (Collet *et al.*, 2012 ▸) and in the excited state structural analyses of several coordination com­plexes displaying microsecond lifetimes (Makal *et al.*, 2012 ▸; Coppens, 2017 ▸).

### Picosecond and XFEL experiments

**Figure 15:**
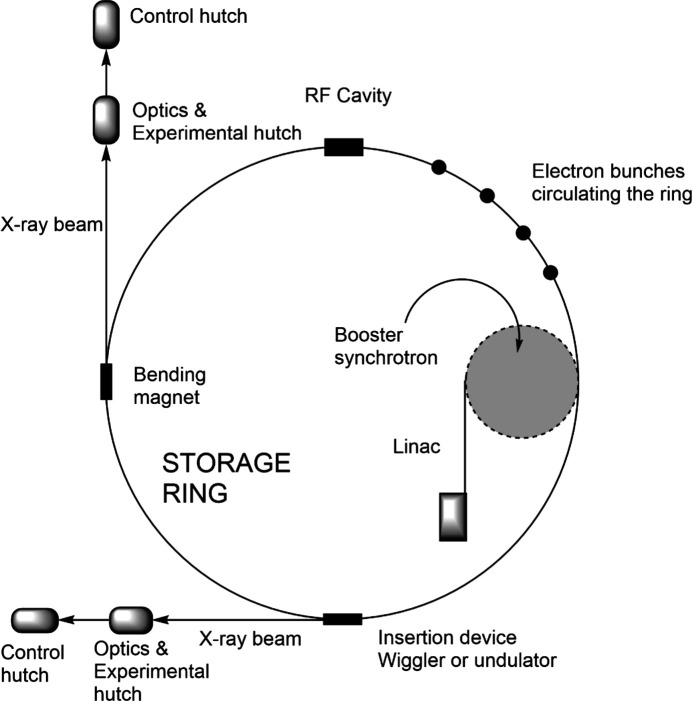
A schematic diagram of a synchrotron showing the various com­ponents.

For excited state species with lifetimes in the picosecond range and below, the time structure of the synchrotron beam itself can be used to provide very short probe pulses of X-rays. A schematic of a synchrotron is illustrated in Fig. 15 ▸ (Clegg, 2000 ▸). The bunch structure of the synchrotron originates from the linear accelerator (Linac). Electrons produced by an electron gun are fired into a Linac and accelerated to nearly the speed of light by a series of oscillating electric potentials along a linear path. The synchronized electric field in the cavities accelerates the electrons each time they pass through, causing them to group into bunches.

The electrons then pass into the booster ring, an ‘athletics track’ containing radio frequency (RF) cavities in the straight sections and bending magnets in the curved sections to keep the electrons circulating. The main purpose of the booster ring is to further accelerate the electrons to the required energy. The megahertz (MHz) oscillating electric field in the RF cavities further enhances the bunches with a separation equal to the frequency of oscillation.

The electron bunches are injected into the main storage ring, which is used to generate electromagnetic radiation for the beamline end stations. Electromagnetic radiation is gen­er­ated when a charged particle, such as an electron bunch, travelling at relativistic speeds is deflected from its original path, resulting in an acceleration when passing through a magnetic field. This acceleration causes the electron bunch to radiate electromagnetic waves (synchrotron light) tangentially to the arc of motion.

The storage ring is a multi-sided polygon with bending magnets at each corner, curving the electron bunches and producing electromagnetic radiation. In the straight sections, insertion devices, arrays of magnets with alternating polarity, force the electron bunches into a sinusoidal path, generating intense electromagnetic radiation.

RF cavities are placed at regular inter­vals around the ring to replenish the energy lost by the generation of electromagnetic radiation. The bunches are synchronized to arrive slightly after the peak of the oscillating electric field, on the downslope of the wave. Electrons within the bunch that have less energy and are, therefore, slightly slower, will arrive later and receive a larger boost, while those with higher energy moving faster will arrive earlier and get a smaller boost. This ensures that bunches remain com­pact and uniformly spaced,

In the storage ring, the time separation between the bunches is determined by the RF cavity frequency. For example, a 500 MHz frequency results in a 2 ns separation between bunches. The orbit time for a single bunch traveling at relativistic speed depends on the ring circumference. At Diamond Light Source (DLS), with a 562 m ring, the orbit time is 1.872 µs, while at Advanced Photon Source (APS), with a 1104 m ring, the orbit time is 3.68 µs. At DLS, with a 500 MHz RF cavity and 2 ns separation, there are 936 available bunch slots. For stability, 900 slots are filled, followed by 36 empty slots, creating a 72 ns gap between the end and start of the main bunch train. For time-resolved experiments, the storage ring can be filled with an arbitrary bunch pattern. One common pattern is the single bunch mode, where only one RF ‘bucket’ is filled. The repetition rate is determined by the storage ring size and the orbit period. At DLS and APS, the repetition rates are 1.871 and 3.68 µs, respectively. APS uses 24 single bunches, giving a 153 ns repetition rate.

The minimum experimental time resolution is determined by the overlap between the X-ray and laser pulses. Intense pulsed lasers used in time-resolved experiments have pulse widths in the femtosecond range. At synchrotrons, X-rays produced by single bunches have pulse widths of a few tens of picoseconds (20–40 ps at DLS and 20–50 ps at APS).

Most synchrotrons can perform pump–probe experiments within the picosecond to nanosecond timeframe without electronic time-gating. The laser pump repetition rate is synchronized with the storage ring repetition rate (Coppens, Iversen & Larsen, 2005 ▸). However, the X-ray flux from a single bunch is much lower than that from a standard fill pattern. At DLS, a single bunch with a charge of 3 nC is com­pared to 900 bunches with a charge of 0.62 nC each. Thus, a single time point would take 186 times longer if the laser repetition rate matches the ring, making these experiments often flux-limited and not feasible for many systems.

To study chemical processes that occur on the femtosecond timescale, more com­plicated experiments need to be designed. For macromolecules, the ultimate approach is the ‘single-shot’ experiment, where the com­plete diffraction pattern from the crystal can be obtained in one X-ray pulse. This is not really feasible for the study of mol­ecular systems because there are only a small number of X-ray reflections on each frame and indexing to obtain a crystallographic unit cell and crystal orientation is difficult. In particular, establishing the intensity of partially recorded diffraction spots relies heavily on the accurate determination of the crystal orientation, which is particularly difficult if there are only a few reflections on a frame, as is the case for mol­ecular crystals with small unit-cell dimensions. The serial femtosecond crystallographic (SFX) approach is employed instead (Barends *et al.*, 2022 ▸), where it is possible to obtain an indexable number of reflections on each frame and then data from many crystals are merged to obtain a com­plete diffraction pattern. Recently, Iversen and co-workers (Støckler *et al.*, 2023 ▸) have developed a data-reduction pipeline approach that automatically handles all the steps in the data-reduction process from spot harvesting to the merging of structure factors. The pipeline uses a sparse indexing approach, based on previously known unit-cell parameters, seed-skewness integration and overlap-based intensity corrections, which can be dynamically adjusted after the initial refinement.

X-ray Free Electron Lasers (XFELs) produce several orders of magnitude higher flux than the most powerful synchrotron and are ideal for studies at the shortest required timescales. However, the experiments are extremely challenging. XFELs have been used to study the dynamic structures of several macromolecular systems using the ‘diffract-and-destroy’ approach, where the crystal is destroyed by the highly intense X-ray pulse, but a diffraction pattern is recorded in a few picoseconds before its destruction (Spence, 2017 ▸; Schmidt, 2019 ▸). In these experiments, repeat measurements are possible if a stream of crystals is passed through the X-ray beam (Nam, 2019 ▸), the serial femtosecond crystallography (SFX) approach (Barends *et al.*, 2022 ▸). In these experiments, a single diffraction image is recorded from an individual crystal with, ideally, enough reflections to allow indexing of the diffraction pattern and, thereby, determination of that crystal’s orientation. The full diffraction pattern is then obtained by merging the data from many tens, if not hundreds, of individual crystals. Inter­est in SFX and other serial crystallography approaches is growing considerably, both in the macromolecular and small mol­ecule disciplines, in line with the continued push to brighter X-ray sources as current ‘third generation’ synchrotron sources are being upgraded (Raimondi *et al.*, 2023 ▸; Watanabe & Tanaka, 2023 ▸), new ‘fourth generation’ synchrotrons are being developed (Chapman, 2023 ▸) and the crystallographic use of XFEL instruments increases. However, SFX experiments are not without significant challenges: scaling between crystals is difficult and it has not been possible to obtain data down to atomic resolution. Furthermore, as mentioned above, the need to obtain enough reflections from a single X-ray shot to enable indexing of the diffraction pattern from a single image is a particular challenge for mol­ecular crystal systems, with typically smaller unit cells and fewer reflections per image. However, the situation changed recently, and Ivsesen and co-workers (Støckler *et al.*, 2023 ▸) have applied their pipeline approach to the previously studied structure of the potassium salt of [Pt_2_(pop)_4_]^4−^ and, using known unit-cell parameters, refined its structure to an *R*_1_ value of *ca* 9.1%. Ihee and co-workers (Kang *et al.*, 2024 ▸) have published a report on the dynamic structure of a mol­ecular metal–organic framework (MOF) mol­ecule using time-resolved serial femtosecond crystallography at the Pohang Accelerator Laboratory X-ray free-electron laser facility. The MOF structure, PCN-224(Fe), consists of a porous coordination network of iron porphyrin units linked with hexazirconium nodes, Zr_6_. The iron centres can bind to carbon monoxide mol­ecules to give the com­plex PCN-224(Fe)-CO, which then undergoes CO loss under photoactivation. The electron-density maps from the study slow loss of the CO ligand after 0.1 ps, together with movement of the iron centre, and these features become more evident after 1.1 ps. Importantly, the atomic resolution of the structures is better than 1 Å, a level of precision that is appropriate for mol­ecular crystallography. Rather than using an injector-style crystal delivery system usually used in macromolecular XFEL studies, Ihee and co-workers chose to employ a fixed-target sample holder, where crystals were placed on a thin film and the film moved between measurements to allow fresh single crystals to be exposed to the pump and probe pulses. This experiment has shown that, with suitable modifications, dynamics within mol­ecular crystals can be studied at very short timescales and sets a benchmark for future studies.

## Conclusions

Since the mid-1990s, with advances in technology and methodology, it is now possible to monitor chemical processes in the crystalline state in real time and obtain structural information on photoactivated mol­ecules with excited state lifetimes from hours to picoseconds using single-crystal X-ray crystallography. Generally, the shorter the lifetime of the mol­ecule whose structure is to be determined, the more com­plex is the Time-Resolved Single-Crystal X-ray Diffraction (TR-SCXRD) experiment. However, with a modern laboratory-based single-crystal X-ray diffractometer fitted with a high-flux microfocus X-ray source and equipped with electronically time-gated pixel detectors, a crystal-cooling apparatus and either a pulsed laser or pulsed LEDs, it is now possible to conduct quite sophisticated TR-SCXRD experiments on mol­ecules that are metastable under the experimental conditions and those with lifetimes down to mil­li­seconds in the home laboratory. For time-resolved studies on mol­ecules with excited state lifetimes in the microsecond to nanosecond range, the use of synchrotron facilities remains the better option since it must be remembered that all TR-SCXRD experiments remain flux-limited, and the lower the X-ray flux, the longer the experiment will take. Pump–probe or pump–multiprobe experiments can be successfully com­pleted using either the time structure of the synchrotron or an electronically time-gated detector to control the probe pulses, and either a pulsed laser or pulsed LEDs to provide the pump pulses. For mol­ecular structures with excited states in the picosecond to femtosecond domain, highly sophisticated XFEL experiments are required. Femtosecond pulsed lasers provide the pump pulses, and the XFEL generates femto- or picosecond X-ray pulses. It is only very recently that XFEL studies on photoactivated mol­ecular materials, with a resolution suitable for an accurate mol­ecular structure determination, have been achieved (Kang *et al.*, 2024 ▸).

Whatever the lifetime of the mol­ecular species being investigated, it is essential to meticulously plan the photocrystallographic experiment before proceeding. If pitfalls in the time-resolved experiment are to be avoided, as much additional information on the reaction as possible needs to be obtained, with a full set of spectroscopic data, thermal analysis, crystal degradation pathways, *etc*. A com­putational analysis of the chemical process is also very helpful, as this information may support the inter­pretation of data from electron-density maps, for example.

What is certain is that the future of TR-SCXRD is exciting. With the recent developments in XFEL studies for mol­ecular materials, serial femtosecond studies will become commonplace (Coppens, 2017 ▸) and transformative new science will result. For the slower timescale processes, in the microsecond to minute lifetime regimes, the development of faster, more efficient, time-gated X-ray detectors and their implementation on laboratory-based X-ray diffractometers will mean that many more ‘routine’ time-resolved experiments will be carried out, without the need to bid for scarce synchrotron time (Coppens, 2015 ▸). The use of multiple techniques to simultaneously monitor solid-state time-resolved processes will become more prominent (Konieczny *et al.*, 2022 ▸; Hasil *et al.*, 2024 ▸) and will assist in the design of new advanced functional materials. For example, the combination of TR-SCXRD with Raman spectroscopy and emission spectroscopy will permit the exploration of mol­ecular processes and transformations within the crystalline environment in ways that have not been possible previously.
